# Allergic Reactions and Immunity in Response to Tick Salivary Biogenic Substances and Red Meat Consumption in the Zebrafish Model

**DOI:** 10.3389/fcimb.2020.00078

**Published:** 2020-03-10

**Authors:** Marinela Contreras, Iván Pacheco, Pilar Alberdi, Sandra Díaz-Sánchez, Sara Artigas-Jerónimo, Lourdes Mateos-Hernández, Margarita Villar, Alejandro Cabezas-Cruz, José de la Fuente

**Affiliations:** ^1^SaBio, Instituto de Investigación en Recursos Cinegéticos IREC-CSIC-UCLM-JCCM, Ciudad Real, Spain; ^2^UMR BIPAR, INRA, ANSES, Ecole Nationale Vétérinaire d'Alfort, Université Paris-Est, Maisons-Alfort, France; ^3^Department of Veterinary Pathobiology, Center for Veterinary Health Sciences, Oklahoma State University, Stillwater, OK, United States

**Keywords:** alpha gal, alpha gal syndrome, tick, zebrafish, allergy

## Abstract

Ticks are arthropod ectoparasite vectors of pathogens and the cause of allergic reactions affecting human health worldwide. In humans, tick bites can induce high levels of immunoglobulin E antibodies against the carbohydrate Galα1-3Galβ1-(3)4GlcNAc-R (α-Gal) present in glycoproteins and glycolipids from tick saliva that mediate anaphylactic reactions known as the alpha-Gal syndrome (AGS) or red meat allergy. In this study, a new animal model was developed using zebrafish for the study of allergic reactions and the immune mechanisms in response to tick salivary biogenic substances and red meat consumption. The results showed allergic hemorrhagic anaphylactic-type reactions and abnormal behavior patterns likely in response to tick salivary toxic and anticoagulant biogenic compounds different from α-Gal. However, the results showed that only zebrafish previously exposed to tick saliva developed allergic reactions to red meat consumption with rapid desensitization and tolerance. These allergic reactions were associated with tissue-specific Toll-like receptor-mediated responses in types 1 and 2 T helper cells (T_H_1 and T_H_2) with a possible role for basophils in response to tick saliva. These results support previously proposed immune mechanisms triggering the AGS and provided evidence for new mechanisms also potentially involved in the AGS. These results support the use of the zebrafish animal model for the study of the AGS and other tick-borne allergies.

## Introduction

Arthropod ectoparasites are a growing burden worldwide (Stutzer et al., 2018). Local allergic reactions to the bite of arthropod ectoparasites such as mosquitoes, ticks, fleas, mites, and lice are common, but in some cases large local and anaphylactic reactions are possible (Lee et al., 2016; Mihara, 2017; Stringer et al., 2017; Haddad et al., 2018; Ha et al., 2019).

Ticks are vectors of pathogens affecting human and animal health worldwide (de la Fuente et al., 2008, 2017). Tick saliva contains multiple biomolecules such as proteins and lipids that facilitate feeding while counteracting host defense responses, properties that also lead to possible application of these molecules in therapeutic interventions (Chmelar et al., 2019). However, tick bites themselves can induce a spectrum of inflammatory reactions in response to toxic and anticoagulant biogenic substances present in tick saliva and/or mouthpart penetration such as coagulative necrosis producing firm papules, tick paralysis, intense pruritus, tick bite alopecia, cutaneous lymphoid hyperplasia, and cell histiocytosis (Mihara, 2017; Stringer et al., 2017; Haddad et al., 2018; Ha et al., 2019). Additionally, tick bites can induce in humans high levels of immunoglobulin E (IgE) antibodies against the carbohydrate Galα1-3Galβ1-(3)4GlcNAc-R (α-Gal) present in glycoproteins and glycolipids from tick saliva that mediate delayed anaphylaxis to red meat consumption, and immediate anaphylaxis to tick bites, xenotransplantation, and certain drugs such as cetuximab (Mateos-Hernández et al., 2017; Hilger et al., 2019). These anaphylactic reactions are known as the alpha-Gal syndrome (AGS) or red meat allergy and are now the focus of recent investigations (Commins et al., 2009; Van Nunen et al., 2009; Platts-Mills et al., 2015; Steinke et al., 2015; Galili, 2018; Cabezas-Cruz et al., 2019; de la Fuente et al., 2019; Hilger et al., 2019).

Recently, C57BL/6 α1,3-galactosyltransferase-knockout (α1,3-GalT-KO) mice that like humans do not synthesize α-Gal have been used as a model to characterize the percutaneous sensitization to α-Gal and *Amblyomma sculptum* tick saliva (Araujo et al., 2016) and the IgE-mediated immune response to cutaneous exposure to *Amblyomma americanum* tick proteins (Chandrasekhar et al., 2019). Additionally, this animal model has been used to study the antibody response to the carbohydrate α-Gal and its potential for the control of infectious diseases caused by pathogens with this modification on their surface (Yilmaz et al., 2014; Cabezas-Cruz et al., 2016; Iniguez et al., 2017; Moura et al., 2017; Portillo et al., 2019). In this context, various fish species constitute models for investigating human diseases (Schartl, 2014), and zebrafish (*Danio rerio* Hamilton 1822) is a relevant animal model for research in genetics, developmental biology, toxicology, oncology, immunology, and allergy (Huang et al., 2018).

In this study, we have developed a new zebrafish animal model for the study of tick-borne allergies caused by biogenic substances present in tick saliva. First, we showed that as occurs in humans, zebrafish do not have α-Gal in their tissues and produce anti–α-Gal IgM antibodies likely in response to bacteria with this modification present in the gut microbiota. Then, an experiment was conducted to evaluate the effect of tick saliva and the salivary components α-Gal and prostaglandin E_2_ (PGE_2_) alone and in combination with red meat consumption on zebrafish allergic response and survival. The results showed that some zebrafish develop hemorrhagic anaphylactic-type reactions provoking deaths in response to tick saliva, but only fish previously exposed to tick saliva develop allergic reactions to red meat consumption with rapid desensitization and tolerance. The immunity in response to tick saliva and red meat consumption showed tissue-specific differences and suggested immune mechanisms triggering the AGS. Taken together, these results identified allergic reactions and immune mechanisms in response to tick saliva and red meat consumption and provided a new animal model for the study of the AGS and other tick-borne allergies.

## Materials and Methods

### Ethics Statement

Animal experiments were conducted in strict accordance with the recommendations of the European Guide for the Care and Use of Laboratory Animals. Animals were housed and experiments conducted at experimental facility (IREC, Ciudad Real, Spain) with the approval and supervision of the Ethics Committee on Animal Experimentation of the University of Castilla La Mancha (PR-2018-06-13) and the Counseling of Agriculture, Environment and Rural Development of Castilla La Mancha (ES130340000218).

### Zebrafish

Wild-type adult (6–8 months old) AB male and female zebrafish were kindly provided by Dr. Juan Galcerán Sáez from the Instituto de Neurociencias (IN-CSIC-UMH, Sant Joan d'Alacant, Alicante, Spain). These zebrafish were certified by Biosait Europe S.L. (Barcelona, Spain; https://biosait.com) as free of major fish pathogens such as *Mycobacterium* spp., *Pseudoloma neurophilia, Pseudocapillaria tomentosa*, and zebrafish retroviruses. The zebrafish were maintained in a flow-through water system at 27°C with a light–dark cycle of 14/10 h and fed twice daily with dry fish feed. For bacterial microbiota studies, 15 freshwater zebrafish adults were also included purchased from a pet store in Ciudad Real, Spain, and used immediately for analysis in the laboratory.

### Zebrafish Feeds and Feeding

Zebrafish were fed before and throughout the experiment twice daily at 9:30 a.m. and 1:30 p.m. Before the beginning of the experiment and up to day 2, all fish were fed with fish feed (Premium food tropical fish, DAPC, Valladolid, Spain; 50–70 μg/fish). On day 2, each experimental group was divided into two subgroups. One subgroup continued to be fed with fish feed at the same regimen, and the second subgroup was fed with dog food (Classic red, ACANA; Champion Petfoods LP, Edmonton, Alberta, Canada; 150–200 μg/fish). The fish feed contains cereals, fish and fish byproducts, soya, yeast, crustaceans, and algae. The dog food is composed of lamb meat meal (23%), steel-cut oats (22%), fresh ranch-raised beef (5%), fresh Yorkshire pork (5%), lamb fat (5%), whole red lentils, whole green peas, whole green lentils, raw grass-fed lamb (4%), whole oats, fresh beef liver (2%), pork meat meal (2%), herring oil (2%), fresh pork liver (2%), whole garbanzo beans, whole yellow peas, sun-cured alfalfa, lentil fiber, fresh beef tripe (1%), dried brown kelp, fresh pumpkin, fresh butternut squash, fresh parsnips, fresh green kale, fresh spinach, fresh carrots, fresh Red Delicious apples, fresh Bartlett pears, freeze-dried beef liver (0.1%), fresh cranberries, fresh blueberries, chicory root, turmeric root, milk thistle, burdock root, lavender, marshmallow root, and rosehips.

### Tick Saliva and Salivary Biogenic Components

*Rhipicephalus sanguineus* (Latreille 1806) female ticks were collected in an animal shelter at Ciudad Real, Spain, while feeding on naturally infested dogs. Ticks were collected at different feeding times for saliva collection as previously described but using pilocarpine hydrochloride (Poole et al., 2013). Partially fed ticks were inoculated with 5 μL of a 2% (wt/vol) solution of pilocarpine hydrochloride in phosphate-buffered saline (PBS), pH 7.4 (Sigma-Aldrich, St. Louis, MO, USA), into the hemocoel using a 50-μL syringe with a 0.33-mm needle (Hamilton Bonaduz AG, Bonaduz, Switzerland). Saliva was harvested using a micropipette, kept on ice, pooled, and stored at −80°C. Saliva protein concentration (1.96 μg/mL) was determined using a BCA Protein Assay Kit (Thermo Fisher Scientific, Waltham, MA, USA) following manufacturer's recommendations. Prostaglandin E_2_ was obtained from Sigma-Aldrich. The bovine serum albumin (BSA) coated with α-Gal (thereafter named α-Gal) was obtained from Dextra (NGP0203 Gala1-3Gal-BSA 3 atom spacer; Shinfield, UK).

### Protein Extracts From Zebrafish Tissues and Feeds, Human HL60 Cells, Pork Kidney, and Tick Salivary Glands

#### Zebrafish, HL60 Cells, and Pork Kidney

Wild-type adult AB zebrafish (*N* = 5; three females and two males) were dissected and muscle, liver/kidney, and gut collected for protein extraction. Human promyelocytic leukemia HL60 cells (ATCC CCL-240; α-Gal negative) were cultured in RPMI 1640 medium supplemented with 10% heat-inactivated fetal calf serum, 2 mM l-glutamine, and 25 mM HEPES buffer as previously described (de la Fuente et al., 2005). Pork (*Sus scrofa*) kidney (1 g; α-Gal positive) was obtained from a slaughterhouse at Ciudad Real, Spain. All samples were homogenized in lysis buffer (7 M urea, 2 M thiourea, 2% 3-[(3-cholamidopropyl)dimethylammonio]-1-propanesulfonate, CHAPS) supplemented with complete mini protease inhibitor cocktail (Roche, Basel, Switzerland). Samples were boiled for 2 min, mixed in a thermocycler for 1 h, and sonicated for 1 min in an ultrasonic cooled bath followed by 10-s vortex. After three cycles of sonication vortex, the homogenate was centrifuged at 200 g for 5 min at 4°C, and the supernatant was quantified using an RC DC protein assay (BioRad, Hercules, CA, USA) with BSA as standard. This methodology has been previously shown to preserve the presence of the α-Gal epitope in extracted proteins (Lima-Barbero et al., 2019).

#### Tick Salivary Glands, Dog Food, and Fish Feed

Salivary glands were dissected from unfed and partially fed *R. sanguineus* female ticks and pooled for analysis (*N* = 10 per pool). Dog food and fish feed were pooled (1 μg per sample) for analysis. Samples were pooled in 500 μL lysis buffer (PBS, 1% Triton X-100) supplemented with complete protease inhibitor mixture (Roche) and homogenized by passing through a needle (27-gauge). Samples were sonicated for 1 min in an ultrasonic cooled bath, followed by vortexing for 10 s. After three cycles of sonication vortex, total protein extracts were centrifuged at 200 g for 5 min to remove debris. The supernatants were collected, and protein concentration was determined using the BCA Protein Assay (Life Technologies, Carlsbad, CA) with BSA as standard following the manufacturer's recommendations.

### Determination of α-Gal Content by Enzyme-Linked Immunosorbent Assay

The α-Gal levels were determined by enzyme-linked immunosorbent assay (ELISA) in zebrafish proteins from different organs, *R. sanguineus* saliva and salivary gland proteins, fish feed, and dog food in comparison with pork kidney (α-Gal–positive control) and human HL60 cells (α-Gal–negative control). Plates were coated with 100 ng proteins per well from different samples in carbonate/bicarbonate buffer incubated overnight at 4°C, following five washes with PBS containing 0.05% Tween 20 (PBST). Unspecific unions were blocked with 1% human serum albumin (HSA; Sigma-Aldrich) and the α-Gal epitope monoclonal antibodies (M86; Enzo Life Sciences, Farmingdale, NY, USA) were added at 1:50 dilution in PBS and incubated for 1 h at 37°C followed by five washes with PBST. Finally, anti–mouse IgM (μ-chain specific)–peroxidase antibody produced in goat (Sigma-Aldrich) was added at 1:2,000 dilution in PBS. Reactions were visualized by adding 100 μL of 3,3′,5,5-tetramethylbenzidine (TMB; Promega, Madison, WI, USA) and incubated for 20 min in the dark at room temperature (RT). The optical density (OD) was measured at 450 nm with an ELISA reader. The average value of the blanks (wells without sample proteins; *N* = 5) was subtracted from all reads, and the average of nine replicates for each sample was used for analysis. A calibration curve with 0.0 to 1.0 ng α-Gal and OD values at 450 nm was constructed using Microsoft Excel for Mac (v. 16.26) to convert ELISA reader values to α-Gal content per sample (*R*^2^ = 0.992; Supplementary Figure 5A). To further validate the calibration curve, a correlation was constructed between 0.0 to 3.5 ng α-Gal and 0.0 to 1.0 μg tick salivary gland proteins using Microsoft Excel for Mac (v. 16.26) (*R*^2^ = 0.992; Supplementary Figure 5B). The results (average ± SD of α-Gal/1 μg protein) were compared between samples and negative or positive controls by Student *t*-test with unequal variance (*p* < 0.05, *N* = 3–5 biological replicates).

### Characterization of α-Gal–Positive Bacteria Zebrafish Gut Microbiota

The study was performed using wild-type adult AB and pet store adult female and male zebrafish (*N* = 5 for each fish group; three females and two males). The microbiota was sampled as previously described (Cantas et al., 2012). Briefly, the ventral belly surface of freshly euthanized fish was opened with sterilized micro–surgical blade and forceps under a light source. The intestinal system was transferred to 1.5-mL tubes containing 200 μL sterile PBS. The intestines were homogenized with a motorized pestle, and disposable plastic loops were used to streak on 5% chicken (α-Gal negative) (Parmentier et al., 2008) blood agar (Rockland Immunochemicals Inc., Pottstown, PA, USA) and tryptic soy agar (Sigma-Aldrich) bacteriological plates for isolation of aerobic and anaerobic bacteria, respectively, following four serial dilutions. The plates were incubated at 28°C and followed by inspections every day for up to 1 week. Bacterial colonies were morphologically classified as aerobic types I (circular, pink, raised punctiform colonies), II (circular, diameter ≤ 5 mm, creamy white, raised colonies), III (irregular, dry white, flat colonies), and anaerobic types Ib (circular, diameter ≤ 5 mm, creamy white, raised colonies) and IIb (circular, white, raised, punctiform colonies). Bacteria isolated from the zebrafish gut microbiota were washed in PBS, fixed, and permeabilized with the Intracell fixation and permeabilization kit (Immunostep, Salamanca, Spain) following manufacturer recommendations. The cells were incubated with 3% HSA (Sigma-Aldrich) in PBS for 1 h at RT. Then, cells were incubated for 14 h at 4°C with the anti–α-Gal monoclonal antibody (M86; Enzo Life Sciences) diluted 1:50 in 3% HSA/PBS. Fluorescein isothiocyanate (FITC) goat anti–mouse IgM (Abcam, Cambridge, UK)–labeled antibody diluted 1:200 in 3% HSA/PBS was used as a secondary antibody and incubated for 1 h at RT. The *Escherichia coli* O86:B7 (ATCC 12701) and BL21 (DE3) cells were included as positive and negative α-Gal controls, respectively (Cabezas-Cruz et al., 2017c). Samples were analyzed on a FACScalibur flow cytometer equipped with CellQuest Pro software (BD BioSciences, Madrid, Spain). The viable cell population was gated according to forward-scatter and side-scatter parameters. The mean fluorescence intensity (MFI) was determined by flow cytometry, and the geometric mean compared between aerobic and anaerobic bacteria by Student *t*-test with unequal variance (*p* = 0.05, *N* = 5 biological replicates).

### Zebrafish Treatment With Tick Saliva and Salivary Biogenic Components

The first trial (Experiment 1) was designed and performed to evaluate the allergic reactions and immune response in zebrafish treated with tick saliva and salivary components and in response to red meat consumption (**Figure 3A**). Adult zebrafish were randomly distributed into five gender-balanced groups (tick saliva, α-Gal, PGE_2_, α-Gal + PGE_2_, PBS) (**Figure 3A**, Table 1). Fish were intramuscularly injected at days 1, 3, and 8 with a Monoject insulin syringe fitted with a 1-cm, 29-gauge needle at the muscle close to the caudal fin with 2.5 μL *R. sanguineus* saliva in 10 μL PBS (tick saliva), 5 μg α-Gal in 10 μL PBS (α-Gal), 350 pg PGE_2_ in 10 μL PBS (PGE_2_), 5 μg α-Gal and 350 pg PGE_2_ in 10 μL PBS (α-Gal + PGE_2_), and 10 μL PBS (PBS). On day 2, each experimental group was randomly divided into two subgroups allocated in two separate water tanks and continued to be fed with fish feed or changed to dog food until the end of the experiment at day 14 when all surviving fish were euthanized (**Figure 3A**, Table 1). Zebrafish local allergic reactions and behavior were examined immediately after treatment or feed change and followed daily until the end of the experiment at day 14. After fish death or euthanize, serum was collected from each animal to determine anti–α-Gal and antitick salivary gland protein IgM antibody titers. Fish were then divided into two longitudinal halves. One-half was used to dissect intestine and kidney for RNA extraction to characterize the mRNA levels for selected immune response markers–correlates of allergy. The second half was used for histochemical characterization of local basophils. Accumulated zebrafish survival was analyzed by a Cox proportional survival regression test (http://statpages.info/prophaz.html) (*p* = 0.05; N = 7–9 biological replicates). Accumulated zebrafish allergy was analyzed by a one-way analysis of variance (ANOVA) test (https://www.socscistatistics.com/tests/anova/default2.aspx) (*p* = 0.05; N = 7–9 biological replicates). The risk of allergic reactions was analyzed in female and male zebrafish by McNemar test (https://www.graphpad.com/quickcalcs/McNemar1.cfm) (*p* = 0.05; *N* = 7–9).

**Table 1:** Experiment 1 design and records of zebrafish allergies and deaths.

**Group**	**Fish No**.	**Gender**	**Feed**	**Day**													
				**1^a^**	**2^b^**	**3^a^**	**4**	**5**	**6**	**7**	**8^a^**	**9**	**10**	**11**	**12**	**13**	**14**
Saliva	26-1	Female	Fish	A	D	Dead											
	26-2	Female	Fish	A	D	Dead											
	26-3	Female	Fish	A	D	Dead											
	26-4	Female	Fish	—	—	—	—	—	—	—	—	—	—	—	—	—	E
	26-5	Male	Fish	—	—	—	—	—	—	—	—	—	—	—	—	—	E
	26-6	Male	Fish	—	—	—	—	—	—	—	—	—	—	—	—	—	E
	14-7	Female	Dog	—	—	A	A	—	—	—	—	—	—	—	—	—	E
	14-8	Male	Dog	—	—	A	A	—	—	—	—	—	—	—	—	—	E
	14-9	Female	Dog	—	—	A	A	—	—	—	—	—	—	—	—	—	E
α-Gal	27-1	Male	Fish	—	—	—	—	—	—	—	—	—	—	—	—	—	E
	27-2	Female	Fish	—	—	—	—	—	—	—	—	—	—	—	—	—	E
	27-3	Female	Fish	—	—	—	—	—	—	—	—	—	—	—	—	—	E
	27-4	Male	Fish	—	—	—	—	—	—	—	—	—	—	—	—	—	E
	15-5	Male	Dog	—	—	—	—	—	—	—	—	—	—	—	—	—	E
	15-5	Male	Dog	—	—	—	—	—	—	—	—	—	—	—	—	—	E
	15-7	Female	Dog	—	—	—	—	—	—	—	—	—	—	—	—	—	E
PGE_2_	28-1	Female	Fish	—	—	—	—	—	—	D	Dead						
	28-2	Female	Fish	—	—	—	—	—	—	—	—	—	—	—	—	—	E
	28-3	Male	Fish	—	—	—	—	—	—	—	—	—	—	—	—	—	E
	28-4	Male	Fish	—	—	—	—	—	—	—	—	—	—	—	—	—	E
	16-5	Female	Dog	—	—	—	—	—	—	—	—	—	—	—	—	—	E
	16-6	Male	Dog	—	—	—	—	—	—	—	—	—	—	—	—	—	E
	16-7	Male	Dog	—	—	—	—	—	—	—	—	—	—	—	—	—	E
α-Gal + PGE_2_	29-1	Female	Fish	AD	Dead												
	29-2	Male	Fish	—	—	—	—	—	—	—	—	—	—	—	—	—	E
	29-3	Male	Fish	—	—	—	—	—	—	—	—	—	—	—	—	—	E
	29-4	Female	Fish	—	—	—	—	—	—	—	—	—	—	—	—	—	E
	29-5	Male	Fish	—	—	—	—	—	—	—	—	—	—	—	—	—	E
	17-6	Male	Dog	—	—	—	—	—	—	—	—	—	—	—	—	—	E
	17-7	Male	Dog	—	—	—	—	—	—	—	—	—	—	—	—	—	E
	17-8	Female	Dog	Disappeared from the tank													
PBS	30-1	Female	Fish	—	—	Died from injection											
	30-2	Female	Fish	—	—	—	—	—	—	—	—	—	—	—	—	—	E
	30-3	Male	Fish	—	—	—	—	—	—	—	—	—	—	—	—	—	E
	30-4	Male	Fish	—	—	—	—	—	—	—	—	—	—	—	—	—	E
	18-5	Female	Dog	—	—	—	—	—	—	—	—	—	—	—	—	—	E
	18-6	Male	Dog	—	—	—	—	—	—	—	—	—	—	—	—	—	E
	18-7	Male	Dog	—	—	—	—	—	—	—	—	—	—	—	—	—	E

a *Treatments according to the experimental group were done at days 1, 3, and 8*.

b *Feed change (fish feed to dog food for some animals) occurred at day 2. The appearance of allergic reactions (A) and death due to allergic reactions (D) were recorded. The absence of allergic reactions and deaths are represented with a dash (—). All surviving fish were euthanized (E) at the end of the experiment*.

A second trial (Experiment 2) was conducted with 10 zebrafish per group and treated with tick saliva and PBS control (**Figure 3B**). Experiment 2 was conducted to inject fish with less amount of tick saliva than in Experiment 1 (1 μL instead of 2.5 μL *R. sanguineus* saliva) to reduce response to toxic and anticoagulant biogenic compounds different from α-Gal and PGE_2_ present in tick saliva and to better monitor the incidence of allergic reactions, abnormal behavior patterns, and feeding during the experiment. As in Experiment 1, adult zebrafish were randomly distributed into two gender-balanced groups (tick saliva PBS) (**Figure 3B**). Fish were intramuscularly injected at days 1, 3, and 8 as in Experiment 1 with 1 μL *R. sanguineus* saliva in 10 μL PBS (tick saliva) and 10 μL PBS (PBS) as control. On day 2, each experimental group was randomly divided into two subgroups (*N* = 5 each) allocated in two separate water tanks and continued to be fed with fish feed or changed to dog food until the end of the experiment at day 10 when all surviving fish were euthanized (**Figure 3B**). Zebrafish local allergic reactions and behavior were examined immediately after treatment or feed change and followed daily until the end of the experiment at day 10. After fish were euthanized, serum was collected from each animal to determine anti–α-Gal IgM antibody titers. The percent of zebrafish affected by allergic reactions and abnormal behavior and feeding on each group fed with fish feed or dog food was compare between saliva-treated and PBS-treated control fish by a one-way ANOVA test (https://www.socscistatistics.com/tests/anova/default2.aspx) (*p* = 0.05; *N* = 4–5 biological replicates).

### Anti–α-Gal IgM Antibody Titers in Zebrafish

For ELISA, high-absorption-capacity polystyrene microtiter plates were coated with 100 ng of α-Gal per well in carbonate–bicarbonate buffer (Sigma-Aldrich). After an overnight incubation at 4°C, coated plates were washed one time with 100 μL/well PBST (Sigma-Aldrich) and then blocked with 100 μL/well of 1% HSA (Sigma-Aldrich) for 1 h at RT. Serum peritoneal fluid samples were diluted (1:100, vol/vol) in blocking solution, and 100 μL/well was added into the wells of the antigen-coated plates and incubated for 1.5 h at 37°C. Plates were washed three times with PBST, and 100 μL/well of species-specific rabbit anti–zebrafish IgM antibodies diluted (1:1,000, vol/vol) in blocking solution was added and incubated for 1 h at RT. Plates were washed three times with 300 μL/well of PBST. A goat anti–rabbit IgG-peroxidase conjugate (Sigma-Aldrich) was added, diluted 1:3,000 in blocking solution, and incubated for 1 h at RT. After four washes with 100 μL/well of PBST, 100 μL/well of TMB (Promega) was added and incubated for 15 min at RT. Finally, the reaction was stopped with 50 μL/well of 2 N H_2_SO_4_, and the OD was measured in a spectrophotometer at 450 nm. The OD at 450 nm was compared between fish treated with saliva, α-Gal, PGE_2_, or α-Gal + PGE_2_, and the PBS-treated control group by Student *t* test with unequal variance (*p* = 0.005; *N* = 7–9). A Spearman ρ correlation analysis (https://www.socscistatistics.com/tests/spearman/Default2.aspx) was performed between anti–α-Gal IgM antibody levels and allergic reactions to tick saliva rated as 10 for fish with allergic reactions and death (AD), 8 for fish with allergic reactions only (A), and 0 for fish without reactions (NR), ρ = 0.179, two-tailed *p* = 0.283.

### Anti-tick Salivary Gland Proteins IgM Antibody Titers in Zebrafish

Proteins were extracted from *R. sanguineus* salivary glands as described above. For ELISA, high-absorption-capacity polystyrene microtiter plates were coated with 100 ng of protein extracts of salivary glands per well in carbonate–bicarbonate buffer (Sigma-Aldrich). After an overnight incubation at 4°C, coated plates were washed one time with 100 μL/well PBST (Sigma-Aldrich) and then blocked with 100 μL /well of 2% BSA (Sigma-Aldrich) for 1 h at RT. Serum peritoneal fluid samples were diluted (1:100, vol/vol) in blocking solution, and 100 μL/well was added into the wells of the antigen-coated plates and incubated for 1.5 h at 37°C. Plates were washed three times with PBST and 100 μL/well of species-specific rabbit anti–zebrafish IgM antibodies diluted (1:2,000, vol/vol) in blocking solution were added and incubated for 1 h at RT. Plates were washed three times with 100 μL/well of PBST. A goat anti–rabbit IgG–horseradish peroxidase conjugate (Sigma-Aldrich) was added diluted 1:3,000 in blocking solution and incubated for 1 h at RT. After four washes with 100 μL/well of PBST, 100 μL/well of TMB solution (Promega) was added and incubated for 10 min at RT. Finally, the reaction was stopped with 50 μL/well of 2 N H_2_SO_4_ and the OD measured in a spectrophotometer at 450 nm. A Student *t*-test with unequal variance was used to compare the OD at 450 nm of IgM antibody titers against tick salivary gland proteins between fish treated with saliva, α-Gal, PGE_2_, or α-Gal + PGE_2_, and the PBS-treated control group (*p* = 0.05; *N* = 7–9) and between zebrafish fed with fish feed or dog food (*p* = 0.05; *N* = 3–6).

### Expression of Selected Immune Response Markers by Quantitative Reverse Transcription–Polymerase Chain Reaction

Total RNA was extracted from zebrafish intestine and kidney samples using the AllPrep DNA/RNA/Protein (Qiagen, Hilden, Germany). The expression of selected zebrafish immune response and food allergy markers (Lu et al., 2008; Huang et al., 2018) *akirin 2* (*akr2*), *complement component c3a* (*c3a*), *interleukin 1-beta* (*il1b*), *interleukin* 4 (*il4*), *nuclear factor interleukin 3 regulated* (*nfil3*), *Toll-like receptor 4b* (*tlr4b*), *interferon-induced GTP-binding protein MxA* (*mxa*), *interferon* (*ifn*), and *MYD88 innate immune signal transduction adaptor* (*myd88*) was analyzed by quantitative reverse transcription–polymerase chain reaction (qRT-PCR) with gene-specific primers (Supplementary Table 1) using the KAPA SYBR FAST one-step universal kit (Sigma-Aldrich) in the Rotor-Gene Q (Qiagen) thermocycler following manufacturer's recommendations. A dissociation curve was run at the end of the reactions to ensure that only one amplicon was formed and that the amplicon denatured consistently in the same temperature range for every sample (Ririe et al., 1997). The mRNA Ct values were normalized against *D. rerio glyceraldehyde-3-phosphate dehydrogenase* (*gapdh*) using the genNormddCT method (Livak and Schmittgen, 2001). The normalized Ct values were compared between fish treated with saliva, α-Gal, PGE_2_, or α-Gal + PGE_2_, and the PBS-treated control group and between fish treated with saliva presenting anaphylactic-type reactions and dead on day 2 and fish without reactions by Student *t*-test with unequal variance (*p* = 0.005; *N* = 3–6).

### Histochemistry of Local Granulocytes in Zebrafish

Euthanized fish at day 14 were sagittal sectioned and then immediately fixed in 10% neutral buffered formalin for 24 h at 21°C, dehydrated in a graded series of ethanol, immersed in xylol, and embedded in paraffin wax using an automatic processor. Sections were cut at 4 mm and stained with hematoxylin and eosin (Sigma-Aldrich) following manufacturer's instructions and standard procedures (Bennett et al., 2001). Stained tissue sections were examined by light microscopy to count granulocytes (three sections of 40 mm^2^ each per fish) and photographed at 40× and 100× magnifications. The average counts of granulocytes were compared between fish treated with tick saliva, α-Gal, or PGE_2_ α-Gal + PGE_2_, and PBS-treated controls and between fish fed on dog food or fish feed for each treatment by Student *t*-test with unequal variance (*p* = 0.05; *N* = 3–6).

## Results

### Zebrafish Do Not Produce α-Gal and Have Natural Anti–α-Gal Antibodies in Response to Bacteria in the Gut Microbiota

This study was designed to evaluate the allergic reactions and immunity in response to tick saliva and salivary biogenic substances such as α-Gal and PEG_2_ and red meat consumption in the zebrafish model.

**Figure 1:**
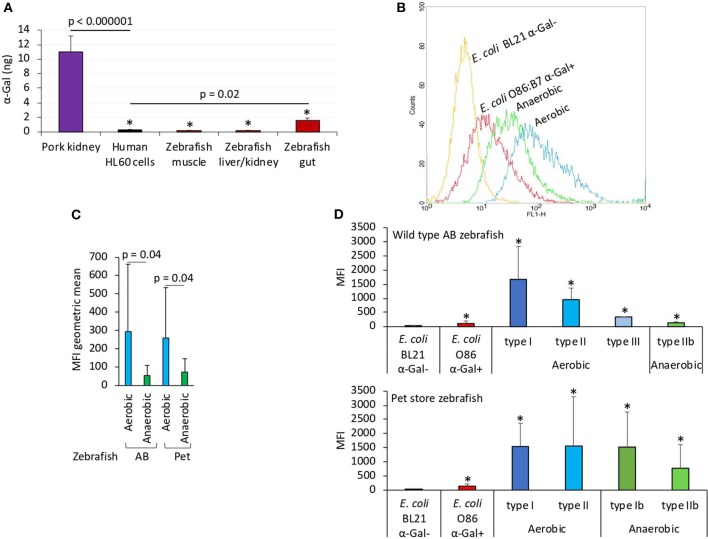
The α-Gal content is similar in humans and zebrafish. The α-Gal content was determined in zebrafish tissues and gut bacterial microbiota and in *R. sanguineus* salivary glands. **(A)** The α-Gal levels were determined by ELISA in zebrafish muscle, liver/kidney, and gut and in comparison with pork kidney (α-Gal positive) and human HL60 cells (α-Gal negative) as positive and negative controls, respectively. The results were converted to α-Gal content per sample using a calibration curve (*R*^2^ = 0.992; Supplementary Figure 5A) and compared between all samples and negative (lines) or positive (^*^p < 1E-8) controls by Student *t*-test with unequal variance (*p* < 0.05, *N* = 5 biological replicates). **(B)** Flow cytometry showing the presence of α-Gal on the surface of aerobic and anaerobic bacteria isolated from zebrafish gut microbiota. *Escherichia coli* O86:B7 and BL21 (DE3) strains were included as positive and negative controls for α-Gal, respectively. For flow cytometry, cells were stained with *Bandeiraea simplicifolia* I-isolectin B4–FITC to visualize α-Gal, and the viable cell population was gated according to forward-scatter and side-scatter parameters. **(C)** The MFI was determined by flow cytometry, and the geometric mean ± SD compared between aerobic and anaerobic bacteria by Student *t*-test with unequal variance (*p* = 0.05, *N* = 5 biological replicates). **(D)** Distribution of the MFI among aerobic and anaerobic type bacteria in wild-type AB and pet store zebrafish and in comparison with *E. coli* O86:B7 and BL21 (DE3)–positive and –negative controls for α-Gal, respectively. The results (average ± SD) were compared between all samples and negative control by Student *t*-test with unequal variance (^*^p < 0.05, *N* = 5 biological replicates).

**Figure 2:**
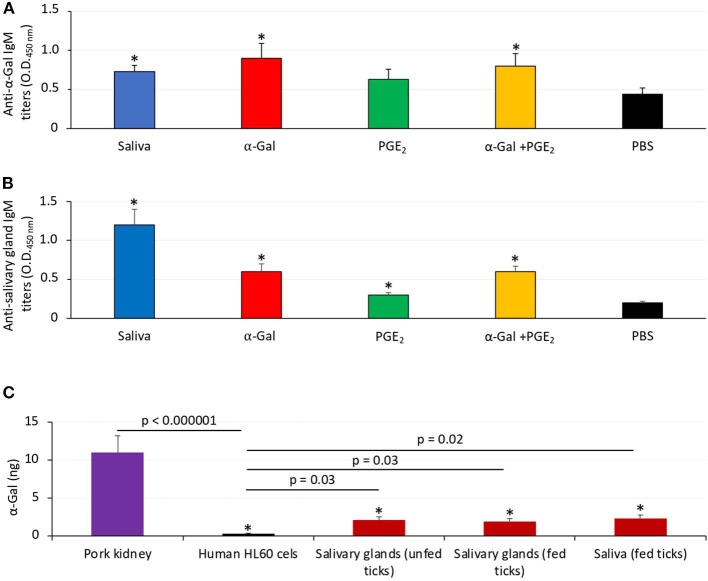
Zebrafish develop antibodies against tick α-Gal and proteins. **(A)** The IgM antibody titers against α-Gal were determined by ELISA, represented as the average ± SD OD at 450 nm and compared between fish treated with saliva, α-Gal, PGE_2_, or α-Gal + PGE_2_, and the PBS-treated control group by Student *t*-test with unequal variance (^*^p < 0.005; *N* = 7–9). **(B)** The IgM antibody titers against tick salivary gland proteins were determined by ELISA, represented as the average ± SD OD at 450 nm and compared between fish treated with saliva, α-Gal, PGE_2_, or α-Gal + PGE_2_ and the PBS-treated control group by Student *t*-test with unequal variance (^*^p < 0.001; *N* = 7–9). **(C)** The α-Gal levels were determined by ELISA in salivary glands from unfed and partially fed ticks and saliva from fed ticks in comparison with pork kidney (α-Gal positive) and human HL60 cells (α-Gal negative) as positive and negative controls, respectively. The results were converted to α-Gal content per sample using a calibration curve (*R*^2^ = 0.992; Supplementary Figure 5A) and compared between all samples and negative (lines) or positive (^*^p < 1E-8) controls by Student *t*-test with unequal variance (*p* < 0.05, *N* = 3 biological replicates).

Herein we first characterized the α-Gal content in fish tissues (Figure 1A). The results showed that only zebrafish gut had α-Gal levels higher than the human HL60 α-Gal–negative control cells, and all zebrafish tissues had significantly lower α-Gal levels than the pork kidney α-Gal–positive control (Figure 1A). Then, the presence of α-Gal was characterized in bacteria from the gut microbiota of laboratory wild-type AB and pet store zebrafish (Figures 1B–D). Identified anaerobic and aerobic gut bacteria had α-Gal levels higher than the *E. coli*—negative and—positive controls (Figure 1B), with higher levels in aerobic than in anaerobic bacteria (Figure 1C). A total of five morphologically different bacterial colonies were isolated in both fish groups with α-Gal content higher than the *E. coli*-negative control (Figure 1D). These results were similar to those described in humans (Galili, 2018) and suggested that natural anti–α-Gal IgM antibody levels in untreated zebrafish are produced in response to gut bacterial microbiota (PBS-treated group; Figure 2A).

Additionally, zebrafish treated with tick saliva, α-Gal, and α-Gal+PGE_2_ developed IgM antibodies against α-Gal that showed higher levels than in fish treated with PGE_2_ or PBS (Figure 2A). Zebrafish treated with tick saliva, α-Gal, PGE_2_, and α-Gal+PGE_2_ but not PBS also developed IgM antibodies against proteins present in tick salivary glands (Figure 2B). Salivary gland proteins in both unfed and partially fed ticks and in tick saliva showed the presence of α-Gal (Figure 2C), thus suggesting that *R. sanguineus* synthesize α-Gal and explaining the anti–α-Gal IgM antibody titers in zebrafish treated with tick saliva (Figure 2A). As expected, because of the presence of PGE_2_ in tick saliva and salivary glands, zebrafish treated with PGE_2_ developed antibodies against salivary gland proteins (Figure 2B) but not against α-Gal (Figure 2A). Finally, a tendency was observed toward higher IgM titers against tick salivary gland proteins in fish fed with dog food when compared to those fed with fish feed (Supplementary Figure 1).

Taken together, these results evidenced similarities in α-Gal content and anti–α-Gal antibody response in zebrafish and humans, suggesting that zebrafish may be evaluated like an animal model for the study of tick-borne allergies produced by salivary biogenic components.

### Zebrafish Develop Hemorrhagic Anaphylactic-Type Reactions and Abnormal Behavior Patterns in Response to Tick Saliva

**Figure 3:**
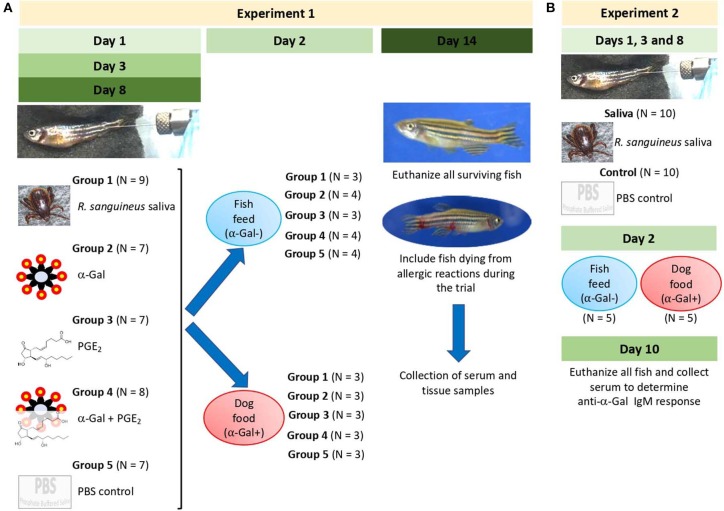
Experimental design. Experiments were designed and performed to evaluate the allergic reactions and immune response in zebrafish treated with tick saliva and salivary components and in response to red meat consumption. **(A)** In Experiment 1, zebrafish were injected with 2.5 μL tick saliva and biogenic substances α-Gal and PGE_2_ to evaluate the allergic reactions and immune response in fish feeding on fish feed or dog food. Serum and tissue samples were collected to determine anti–α-Gal IgM response, intestine and kidney for qRT-PCR analysis of immune response markers, and half fish for histochemical characterization of local granulocytes. **(B)** Experiment 2 was conducted to inject fish with less amount of tick saliva than in Experiment 1 (1 μL instead of 2.5 μL *R. sanguineus* saliva) to reduce response to toxic and anticoagulant biogenic compounds different from α-Gal and PGE_2_ present in tick saliva and to better monitor the incidence of allergic reactions, abnormal behavior patterns, and feeding during the experiment. Zebrafish local allergic reactions and behavior were examined immediately after treatment or feed change and followed daily until the end of the experiment at day 14 (Experiment 1) or day 10 (Experiment 2). Fish and tick representative images are shown.

The study was designed with two experiments to characterize the allergic response in zebrafish exposed to tick saliva and salivary biogenic components such as α-Gal and PGE_2_ (Figures 3A,B). In Experiment 1 (Figure 3A), zebrafish were injected with 2.5 μL tick saliva and biogenic substances α-Gal and PGE_2_ to evaluate the allergic reactions and immune response in fish feeding on fish feed or dog food. Experiment 2 (Figure 3B) was then conducted to inject fish with less tick saliva than in Experiment 1 (1 μL instead of 2.5 μL *R. sanguineus* saliva) to reduce responses to toxic and anticoagulant biogenic compounds different from α-Gal and PGE_2_ present in tick saliva and to better monitor the incidence of allergic reactions, abnormal behavior patterns, and feeding during the experiment.

**Figure 4:**
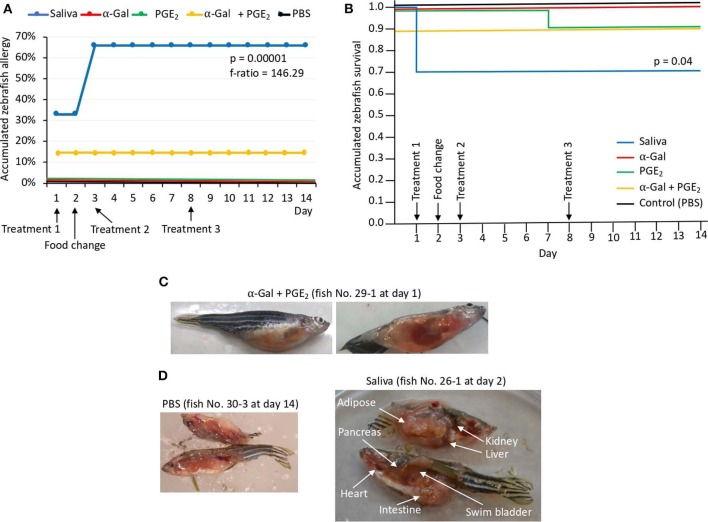
Zebrafish injected with tick saliva develop hemorrhagic anaphylactic-type reactions (Experiment 1). **(A)** Accumulated zebrafish allergy was compared between different groups by a one-way ANOVA test (*p* < 0.05; *N* = 7–9 biological replicates). **(B)** Accumulated zebrafish survival in the different groups was compared by a Cox proportional survival regression test (*p* < 0.05; *N* = 7–9 biological replicates). **(C)** Signs of hemorrhagic anaphylactic-type reactions in zebrafish 29-1 injected with α-Gal + PGE_2_. Fish No. 29-1 died on day 1. **(D)** Representative comparison between necropsied control fish No. 30-1 injected with PBS and fish No. 26-1 injected with tick saliva. Evidence of hemorrhage is shown in organs of a saliva-injected fish.

In Experiment 1, the results showed that the incidence of allergic reactions was statistically significant in zebrafish treated with tick saliva (six animals; 66%) but not in fish treated with α-Gal, PGE_2_, α-Gal + PGE_2_, and PBS (Figure 4A, Table 1). In three animals treated with tick saliva (33%) and before food change, these reactions resulted in death that significantly affected fish survival rate (Figure 4B, Table 1). Although not statistically significant, one fish treated with α-Gal + PGE_2_ also developed allergy and died at day 1 (Figures 4A–C, Table 1). The results showed that zebrafish response to tick saliva was characterized by hemorrhagic anaphylactic-type reactions appearing 3 to 5 h posttreatment (hpt) with hemorrhage affecting various organs (Figure 4D). Although allergy was more prevalent in female than male zebrafish, the analysis by McNemar test of the risk of developing allergic reactions in response to tick saliva did not show a significant association in female (*p* = 0.07) and male (*p* = 1.00) zebrafish.

Abnormal behavior patterns in Experiment 1 consisted of low mobility, permanence at the bottom of the water tank, and zigzag-type swimming (Supplementary Figures 2A–F). Low mobility and permanence at the bottom of the water tank were shown in three zebrafish injected with tick saliva (Supplementary Figure 2A), one zebrafish injected with α-Gal (Supplementary Figure 2C), and one zebrafish injected with α-Gal + PGE_2_ (Supplementary Figure 2E). Normal behavior patterns were seen in all zebrafish injected with PGE_2_ and PBS (Supplementary Figures 2D,F).

These results provided evidence to support that zebrafish develop delayed hemorrhagic anaphylactic-type reactions affecting survival and behavior in response to tick saliva.

### Zebrafish Develop Allergic Reactions and Abnormal Behavior Patterns in Response to Red Meat Consumption After Exposure to Tick Saliva

**Figure 5:**
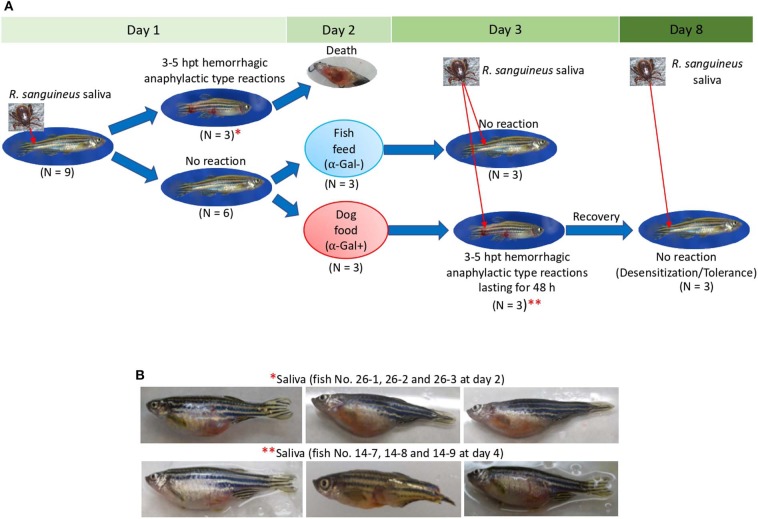
Zebrafish injected with tick saliva and fed with red meat develop hemorrhagic anaphylactic-type reactions and desensitization (Experiment 1). **(A)** In the zebrafish model, 33% of animals treated with tick saliva on day 1 developed hemorrhagic anaphylactic-type allergic reactions 3–5 hpt and died. Furthermore, 100% of fish fed with dog food, but none of the fish that continued feeding on fish feed at day 2, developed allergy to tick saliva injected on day 3. Once recovered from anaphylactic-type reactions, all fish were desensitized and became tolerant to tick saliva injected on day 8. **(B)** Signs of hemorrhagic anaphylactic-type reactions in zebrafish No. 26-1, 26-2, and 26-3 injected with tick saliva and dying at day 2 before food change. After food change, only fish No. 14-7, 14-8, and 14-9 feeding on dog food developed anaphylactic-type reactions. Asterisks in red connect representative fish images in **(A)** with results in **(B)**.

The results of the Experiment 1 (Figure 3A) showed that zebrafish develop delayed hemorrhagic anaphylactic-type reactions and abnormal behavior patterns primarily in response to tick saliva resulting in deaths for 33% of the animals (Figures 5A,B). However, anaphylaxis to consumption of red meat with high α-Gal content is one of the symptoms of the AGS.

**Figure 6:**
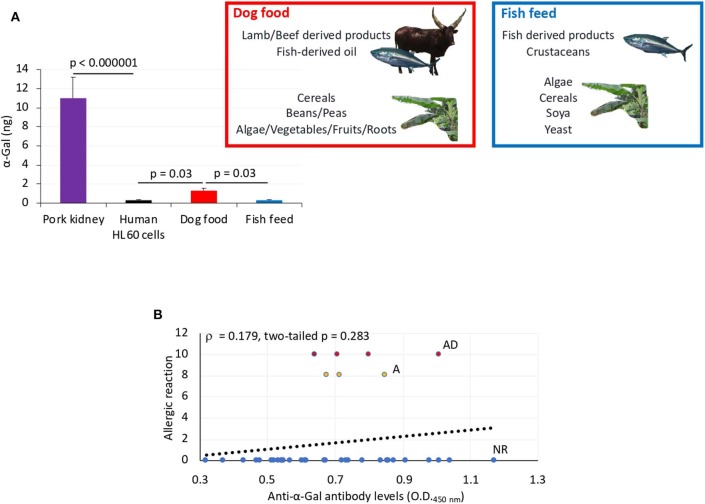
α-Gal levels in dog food and fish feed and correlation analysis between anti–α-Gal IgM antibody levels and allergic reactions to tick saliva. **(A)** The α-Gal levels were determined by ELISA in fish feed and dog food and in comparison with pork kidney (α-Gal positive) and human HL60 cells (α-Gal negative) as positive and negative controls, respectively. The results were converted to α-Gal content per sample using a calibration curve (*R*^2^ = 0.992; Supplementary Figure 5A) and compared between samples and negative control and between dog food and fish feed by Student *t*-test with unequal variance (*p* < 0.05, *N* = 3 biological replicates). The main components of dog food and fish feed are shown. Only dog food contains α-Gal–positive animal-derived products. **(B)** Spearman ρ correlation analysis between anti–α-Gal IgM antibody levels and allergic reactions to tick saliva in Experiment 1 rated as 10 for fish with allergic reactions and death (AD), 8 for fish with allergic reactions only (A), and 0 for fish without reactions (NR). Correlation rank coefficient (ρ) and *p*-value are shown.

To address this sign of the AGS, zebrafish on each treatment group in Experiment 1 and without reactions in response to first treatment with tick saliva on day 1 were split into two subgroups on day 2 (Figures 3A, 5A, Table 1). One subgroup continued to feed on fish feed without α-Gal, whereas the other was fed with dog food containing α-Gal (Figure 6A), and both were treated again with tick saliva on day 3 (Figures 3A, 5A, Table 1). All zebrafish feeding on fish feed did not develop any visible reaction to tick saliva injected on day 3 (Figure 5A, Table 1). However, zebrafish fed with dog food and treated with tick saliva on day 3 did develop delayed (3–5 hpt) hemorrhagic anaphylactic-type reactions that lasted for 48 h (Figures 5A,B, Table 1). An abnormal zig-zag type swimming was also observed at day 3 in fish No. 14-8 injected with tick saliva and fed with dog food (Supplementary Figures 2B, 3). These fish recovered from allergic reactions after 48 h and as animals fed on fish feed did not develop any reactions when treated again with tick saliva on day 8 (Figure 5A). Although a tendency was observed toward a positive correlation between allergic reactions to tick saliva and anti–α-Gal IgM antibody levels, the correlation was not significant (Figure 6B). However, all fish developing anaphylactic-type reactions had IgM antibody levels higher than 0.6 OD at 450 nm (Figure 5C).

**Figure 7:**
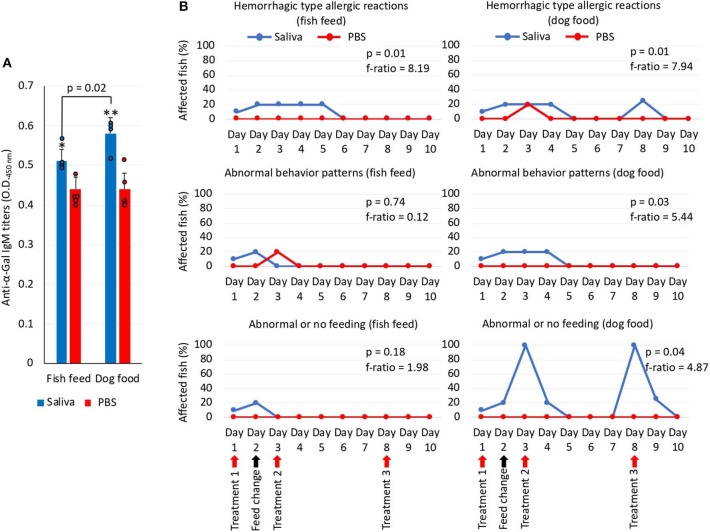
Zebrafish injected with tick saliva and fed with red meat develop allergic reactions and abnormal behavior and feeding patterns (Experiment 2). **(A)** The IgM antibody titers against α-Gal were determined by ELISA, represented as the average ± SD OD at 450 nm and compared between fish treated with tick saliva and the PBS-treated control group and between fish fed on fish feed or dog food Student *t*-test (^*^p = 0.003, ^**^p = 0.0008; *N* = 4–5 biological replicates with individual values shown). **(B)** Zebrafish local allergic reactions and behavior were examined immediately after treatment or feed change and followed daily until the end of the experiment at day 10. The percent of zebrafish affected by allergic reactions and abnormal behavior and feeding on each group fed with fish feed or dog food was compared between saliva-treated and PBS-treated control fish by a one-way ANOVA test (https://www.socscistatistics.com/tests/anova/default2.aspx) (*p* = 0.05; *N* = 4–5 biological replicates).

To gain additional information on the zebrafish allergic reactions and abnormal behavior patterns in response to red meat consumption after exposure to tick saliva, Experiment 2 was conducted (Figure 3B). One fish died on each group on day 5 but for reasons not related to the treatments. Although fish were injected with less tick saliva than in Experiment 1, the anti–α-Gal IgM antibody levels were higher than in PBS-treated controls (Figure 7A). Furthermore, in fish treated with tick saliva but not in controls, the anti–α-Gal IgM antibody levels were higher in fish fed on dog food than in those fed with fish feed (Figure 7A). The analysis of the fish affected by hemorrhagic-type allergic reactions and abnormal behavior and feeding showed that the percentage of affected fish with allergic reactions was higher in saliva-treated than in PBS-treated controls fed with either fish or dog food (Figure 7B). However, these reactions appeared after third treatment with tick saliva only in fish fed on dog food (Figure 7B). Abnormal behavior patterns and feeding were higher in saliva-treated fish than in controls only when feeding on dog food (Figure 7B).

These results showed that zebrafish treated with tick saliva develop hemorrhagic-type allergic reactions with abnormal behavior patterns with higher incidence in fish fed with α-Gal–positive dog food than with α-Gal–negative fish feed. Once recovered from allergic reactions, fish continuing feeding on dog food became tolerant to tick saliva. A risk factor associated with anti–α-Gal IgM antibody levels was also identified.

### Allergic Reactions to Tick Saliva and Red Meat Consumption in Zebrafish Are Associated With Different Tissue-Specific Immune Response Mechanisms

**Figure 8:**
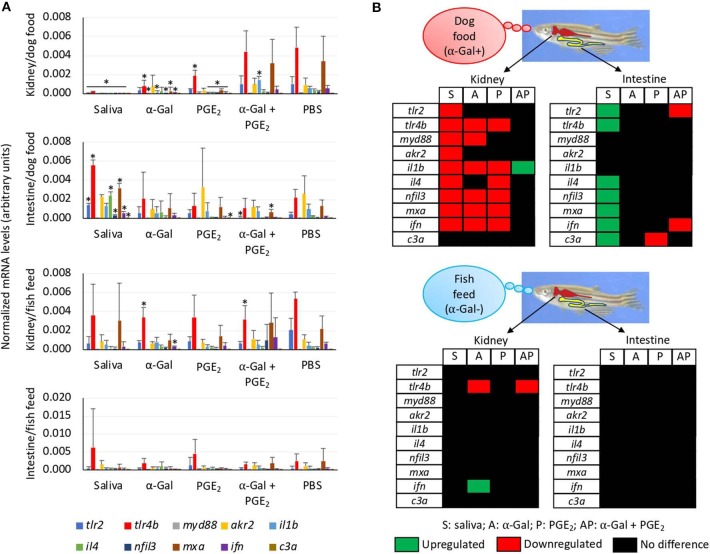
Tissue-specific differences in the immune response of zebrafish injected with tick saliva and fed with red meat (Experiment 1). **(A)** The expression of selected immune response and food allergy markers was analyzed by qRT-PCR in the kidney and intestine of zebrafish fed on dog food or fish feed. The mRNA Ct values were normalized against *D. rerio gapdh*, presented as average ± SD, and compared between fish treated with saliva, α-Gal, PGE_2_, or α-Gal + PGE_2_, and the PBS-treated control group by Student *t*-test with unequal variance (^*^p < 0.05; *N* = 3–6). **(B)** Representation of differential gene expression with respect to PBS-treated controls in the kidney and intestine of zebrafish fed on dog food or fish feed. Data were obtained from the analysis described in **(A)**.

The expressions of selected immune response and food allergy markers were characterized in Experiment 1 using the kidney and intestine involved in both innate and adaptive fish immunity. Different immune responses were observed in zebrafish kidney and intestine and between fish fed on dog food and fish feed (Figures 8A,B). In the kidney of zebrafish fed on dog food but not on fish feed, except for *c3a*, all genes were downregulated in response to tick saliva when compared to PBS-treated controls (Figures 8A,B). However, in the intestine of fish fed on dog food all genes except for *myd88, akr2*, and *il1b* were upregulated in response to tick saliva but not to other treatments (Figures 8A,B). In response to α-Gal or PGE_2_ but not to the combined α-Gal + PGE_2_, various genes were downregulated in the kidney of zebrafish fed on dog food (Figures 8A,B). Minor or no changes in gene expression were observed in the kidney and intestine of fish fed on fish feed and treated with tick saliva, α-Gal, PGE_2_, or α-Gal + PGE_2_ when compared to PBS controls (Figures 8A,B).

**Figure 9:**
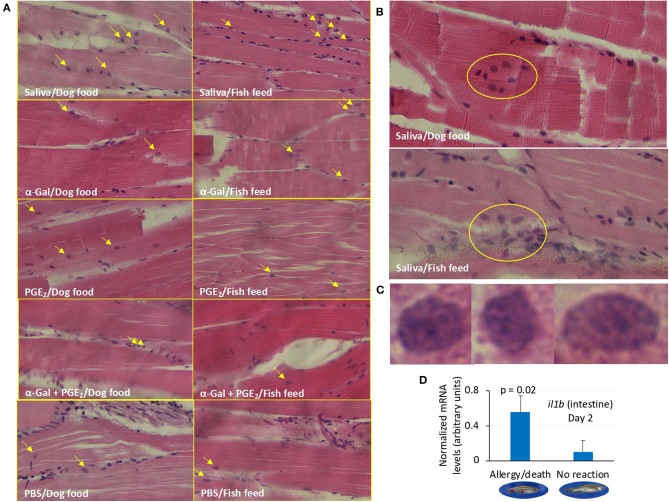
Granulocyte profile in zebrafish (Experiment 1). **(A)** Representative images of granulocytes detected in tissue sections stained with hematoxylin and eosin of zebrafish treated with saliva, α-Gal, PGE_2_, or α-Gal + PGE_2_, or the PBS control and fed with dog food or fish feed. The fields were randomly chosen, and granulocytes are indicated with arrows. The average counts of granulocytes were compared between fish treated with tick saliva, α-Gal, or PGE_2_ α-Gal + PGE_2_, and PBS-treated controls and between fish fed on dog food or fish feed for each treatment by Student *t*-test with unequal variance (*p* < 0.05; *N* = 3–6; Supplementary Figure 4). Most granulocytes were observed in the skeletal muscle. Magnification ×40. **(B)** Representative images of granulocytes agglomerations only detected in zebrafish treated with tick saliva. Magnification ×40. **(C)** Selected images for identified granulocytes showing characteristics of basophils/eosinophils. Magnification ×100. **(D)** The expression of selected immune response and food allergy markers was analyzed by qRT-PCR in the kidney and intestine of zebrafish treated with saliva and presenting anaphylactic-type reactions and death on day 2, and fish without reactions and normalized mRNA Ct values (average ± SD) were compared by Student *t*-test with unequal variance (*p* < 0.05; *N* = 3–6). Only *il1b* gene in the intestine showed significant differences.

The analysis of granulocytes in zebrafish tissue sections collected in Experiment 1 identified the presence of these cells mainly in the skeletal muscle (Figure 9A and Supplementary Figure 4). The results showed a higher number (*p* = 0.00000002) of granulocytes and granulocyte agglomerations in zebrafish treated with tick saliva (8.8 ± 0.8) when compared to fish treated with α-Gal (2.7 ± 0.8), PGE_2_ (3.2 ± 0.8), α-Gal + PGE_2_ (3.2 ± 1.0), or PBS (2.3 ± 0.6) (Figures 9A,B, Supplementary Figure 4). No differences were observed between fish fed on fish feed or dog food for each treatment (Supplementary Figure 4). At a higher magnification, the structure of the granulocytes showed characteristics of fish basophils/eosinophils such as a highly granular cytoplasm with large and spherical granules (Figure 9C).

Finally, of the selected zebrafish immune response and food allergy markers, only the expression of *il1b* was significantly higher in the intestine of zebrafish treated with tick saliva and presenting anaphylactic-type reactions and death when compared to fish without reactions on day 2 (Figure 9D).

These results suggested that tick salivary biogenic components different from or in combination with α-Gal and PGE_2_ are essential for the modulation of zebrafish immune response to tick saliva and red meat consumption in both kidney and intestine but affecting different tissue-specific mechanisms. The results also suggested a role for basophils/eosinophils in zebrafish response to tick saliva.

## Discussion

Tick saliva contains biogenic substances including proteins, lipids, and other biomolecules such as PGE_2_ and α-Gal that modulate multiple biological processes affecting ectoparasite feeding and pathogen infection and transmission (Oliveira et al., 2011; Poole et al., 2013; Chmelar et al., 2019). These molecules may also affect host immune response leading to allergic diseases such as the AGS (Araujo et al., 2016; Cabezas-Cruz et al., 2017c, 2019; Chandrasekhar et al., 2019; Hilger et al., 2019). In this study, we focused on the brown dog tick *R. sanguineus* based on the worldwide distribution of this tick species as a major dog ectoparasite, the risk it poses for urban populations, its role in the transmission of pathogens such as *Rickettsia rickettsii* causing Rocky Mountain spotted fever and the cause of anaphylactic reactions to tick bite, and its phylogenetically close relationship with tick species such as *Rhipicephalus bursa* and *Rhipicephalus microplus* previously shown to contain α-Gal–modified proteins (Valls et al., 2007; Uspensky, 2014; de la Fuente et al., 2017; Mateos-Hernández et al., 2017).

Humans evolved with the inactivation of the α1,3-GalT gene, which resulted in the recognition of the carbohydrate α-Gal as a non–self-antigen, thus inducing the production of high antibody titers against this molecule (Galili, 2018). This evolutionary trait benefits humans by providing immunity to pathogens containing α-Gal in the surface while increasing the risks of developing the AGS triggered by the IgE antibody response against α-Gal present in glycoproteins and glycolipids from tick saliva and tissues of non-catarrhine mammals (Commins et al., 2009; Van Nunen et al., 2009; Platts-Mills et al., 2015; Steinke et al., 2015; Galili, 2018; Cabezas-Cruz et al., 2019; de la Fuente et al., 2019; Hilger et al., 2019; Román-Carrasco et al., 2019; Park et al., 2020). The AGS is characterized by delayed anaphylaxis to red meat consumption and immediate anaphylaxis to tick bites, xenotransplantation, and certain drugs such as cetuximab (Mateos-Hernández et al., 2017; Hilger et al., 2019). Despite recent advances in the study of the AGS (Commins et al., 2009; Van Nunen et al., 2009; Platts-Mills et al., 2015; Steinke et al., 2015; Mateos-Hernández et al., 2017; Galili, 2018; Cabezas-Cruz et al., 2019; de la Fuente et al., 2019; Hilger et al., 2019), the immune-mediated mechanisms induced by tick bites and leading to the AGS have been only partially characterized in α1,3-GalT-KO mice (Araujo et al., 2016; Chandrasekhar et al., 2019). The development of new animal models for tick-borne allergies such as the AGS would contribute to these studies.

Considering that zebrafish are evolutionarily naive to tick saliva as they are not naturally exposed to ticks, the observed allergic hemorrhagic anaphylactic-type reactions and abnormal behavior patterns may occur in response to toxic and anticoagulant biogenic compounds different from α-Gal and PGE_2_ present in tick saliva (Francischetti et al., 2009; Aleman and Wolberg, 2013; Mihara, 2017; Stringer et al., 2017; Haddad et al., 2018). For example, although uncommon, episodic hemorrhage has been described in humans during honeybee venom anaphylaxis (Mingomataj and Bakiri, 2012). This episodic hemorrhage has been associated with honeybee venom components that interfere with complement cleavage and bradykinin release, thus affecting coagulation, thrombolysis, hemolysis, and smooth muscle tone. Additionally, infestations by sea lice of the family Caligidae such as *Lepeophtheirus* and *Caligus* species affect fish behavior and cause abrasion-like lesions at their attachment and feeding sites by changing mucus consistency and damaging the epithelium, which results in loss of blood and fluids and cortisol release (Fast, 2014; Øverli et al., 2014). Some species of fish will spend more time lying on the bottom of the tank when they become stressed, which will also reduce eating (Kalueff et al., 2013). Zigzagging is also a behavior associated with fish stress (Kalueff et al., 2013).

Anaphylactic-type reactions have been previously described in channel catfish (*Ictalurus punctatus* Refinesque) and goldfish (*Carassius auratus* L.) following immunization and challenge with solubilized protozoa (*Tetrahymena pyriformis*) and human serum proteins, respectively, but not when challenged with the heterologous BSA antigen (Goven et al., 1980). The fishes sensitized and challenged with homologous antigens showed abnormal behavior patterns consisting of disorientation, breading problems, and increased defecation. Severe respiratory distress resulted in 33% death of treated catfish. The authors concluded that type I hypersensitivity reactions were the cause of observed anaphylaxis, a mechanism currently defined as type 2 T helper (T_H_2) immunity that has been proposed to be associated with the AGS (Wilson et al., 2017; Cabezas-Cruz et al., 2019; Chandrasekhar et al., 2019). In our study, the potential role of BSA present in in the α-Gal–coated particles in the observed allergic reactions when administered to zebrafish was discarded because as previously described (Goven et al., 1980), fish were not previously exposed to BSA, and BSA was likely not present in the tick saliva. Although fish were intramuscularly injected with tick salivary biogenic substances, part of the injected liquid remained subcutaneous, which would better resemble tick bites.

The effect of red meat consumption in the form of dog food containing α-Gal in zebrafish previously treated with tick saliva and normally fed with fish feed free of α-Gal is relevant for the study of the AGS. Dog feed was used because it contains fish-and plant-derived components also present in fish food, and it is registered for animal use. Both fish feed and dog food contained plant-derived compounds together for either fish or animal (red meat) products, thus making it difficult to assign the observed reactions to other compounds present in dog food. We did not add α-Gal to fish feed because it is possible that the immune response to α-Gal depends on the way this molecule is presented on proteins or lipids and not only the carbohydrate by itself. For still unknown reasons and despite identified risk factors such as gender, pollen allergy, bronchial asthma, pet keeping, age, blood group, and lifestyle (Cabezas-Cruz et al., 2017a,b, 2019), only a fraction of the humans exposed to tick bites develop the AGS, and cases of mammalian meat desensitization have been recently reported (Yucel et al., 2019). Despite the limitations associated with the low number of fish included on each treatment, in the zebrafish model 33% (Experiment 1) and 20% (Experiment 2) of the animals treated with tick saliva developed allergic reactions with no reactions in control fish. However, when fed with dog food, 100% of the animals in both experiments presented allergic reactions including abnormal behavior or feeding after treatment with tick saliva. Furthermore, once recovered from anaphylactic-type reactions, fish became tolerant to tick saliva by still unknown mechanisms. Differences in the presentations of allergic reactions between Experiments 1 and 2 may be due to the amount of tick saliva injected in fish (2.5 μL in Experiment 1 vs. 1 μL in Experiment 2). These results suggested a role for red meat consumption in the allergic reactions to tick saliva in zebrafish and in a rapid desensitization process to become tolerant to tick saliva.

In humans, anti–α-Gal IgE antibody levels (≥0.35 kU/L) have been identified like a risk factor for the development of the AGS (Commins et al., 2009; Van Nunen et al., 2009; Platts-Mills et al., 2015; Steinke et al., 2015; Galili, 2018; Cabezas-Cruz et al., 2019; de la Fuente et al., 2019; Hilger et al., 2019). Recently and albeit that cofactors are influential in the expression of mammalian meat allergy (Platts-Mills et al., 2020), Mabelane et al. (2018) reported that anti–α-Gal IgE antibody levels higher than 5.5 kU/L are an indicator of AGS with 95% confidence. Herein we showed that zebrafish as humans do not synthesize α-Gal and produce natural anti–α-Gal IgM antibodies in response to gut bacterial microbiota. However, in zebrafish, a significant correlation was not observed between anti–α-Gal IgM antibody levels and allergic reactions to tick saliva, but IgM antibody levels higher than 0.6 OD at 450 nm were identified as a risk factor for developing anaphylactic-type reactions to tick saliva and red meat consumption. Nevertheless, these results may be affected by the fact that the antibody levels in zebrafish dying from anaphylactic-type reactions were determined on day 2 after a single treatment with tick saliva, whereas most fish were treated three times and sera collected on day 14 for analysis.

**Figure 10:**
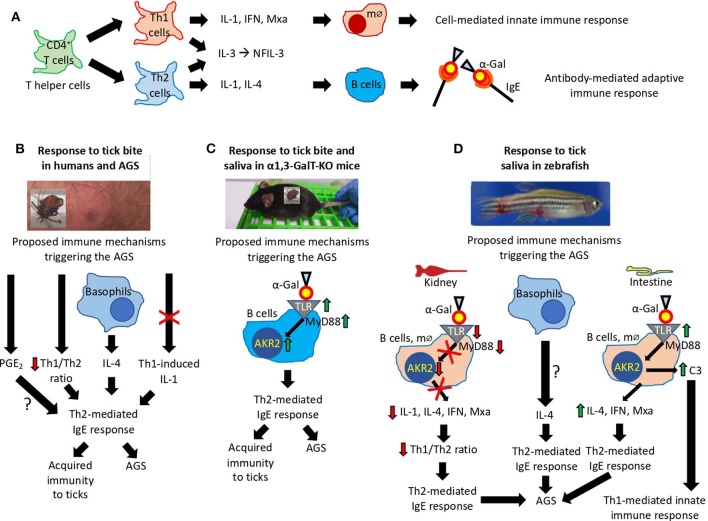
Proposed mechanisms triggering the AGS. **(A)** Mechanisms mediated by CD4^+^ T cells or T helper (T_H_) cells that develop into T_H_1 and T_H_2 cells regulating cell-mediated and antibody-mediated innate and adaptive immune responses, respectively. **(B–D)** Immune mechanisms triggering the AGS that have been proposed based on existing evidence in **(B)** humans, **(C)** α1,3-GalT-KO mice, and **(D)** our zebrafish animal model. The interrogation marks (?) represent mechanisms that need additional evidence to be sustained.

To identify the possible immune mechanisms associated with allergic reactions observed in zebrafish in response to tick saliva and red meat consumption, selected immune response and food allergy markers involved in T_H_1 and T_H_2 cell–mediated responses (Lu et al., 2008; Huang et al., 2018) were characterized in the kidney and intestine involved in both innate and adaptive fish immunity (Liu et al., 2015; Brugman, 2016; Martins et al., 2019). The CD4^+^ T cells or T helper cells develop into T_H_1 and T_H_2 cells (Figure 10A). While type 1 T helper (T_H_1) cells produce interferon (IFN), interelukin 1 (IL-1), and Mxa, among other proteins, for cell-mediated immunity and macrophage-dependent protective responses, T_H_2 cells produce IL-1, IL-4, and other cytokines to induce antibody-mediated adaptive immune response and inhibition of several macrophage functions (Romagnani, 1999). Other cytokines such as IL-3 and regulated factors (NFIL-3) are produced by both T_H_1 and T_H_2 cells (Romagnani, 1999). Additionally, T_H_1 cells are involved in the pathogenesis of organ-specific autoimmune disorders, whereas T_H_2 mediates allergen-specific responses (Romagnani, 1999).

In humans, the AGS has been proposed to be associated with tick saliva–induced inhibition of T_H_1-induced production of IL-1, basophil-mediated production of IL-4, decrease in T_H_1/T_H_2 ratio, and PGE_2_-induced antibody class switching, all resulting in the induction of T_H_2-mediated IgE response against α-Gal (Cabezas-Cruz et al., 2019; Kageyama et al., 2019) (Figure 10B). As recently concluded by Kageyama et al. (2019), repeated tick bites promote basophil recruitment and attract T_H_2 cells to the skin, which results in a proper cytokine milieu to enhance IgE antibody levels against tick proteins and α-Gal to facilitate acquired immunity to ticks and the AGS.

In the α1,3-GalT-KO mouse model, the induction of α-Gal–specific IgE antibodies following tick feeding and in response to subcutaneous injection of tick saliva was proposed to be associated with the salivary proteins modified with α-Gal–like antigens that might modulate host immune response toward anti–α-Gal IgE antibodies (Araujo et al., 2016). However, recently Román-Carrasco et al. (2019) provided evidence supporting that glycolipids but not glycoproteins containing α-Gal were able to cross the intestinal monolayer and trigger an allergic reaction such as the AGS. To characterize the immune mechanisms leading to production of IgE antibodies and allergic reactions in response to tick bites and red meat consumption, Chandrasekhar et al. (2019) showed in the α1,3-GalT-KO mouse model that the induction of IgE responses was dependent on CD4^+^ T cells and the expression of the B cell-intrinsic MyD88 adaptor for inflammatory Toll-like receptor (TLR) and IL-1 signaling pathways leading, among others, to the activation of the Akr2/nuclear factor κB (NF-κB) (Deguine and Barton, 2014) (Figure 10C).

The results reported here in the zebrafish model support some of the previously proposed immune mechanisms triggering the AGS and provided evidence for different tissue-specific mechanisms also potentially involved in the AGS (Figure 10D). While in zebrafish kidney α-Gal–containing glycolipids and glycoproteins may antagonize TLR-mediated response to promote T_H_2-mediated IgE response to α-Gal, in the intestine a mechanism similar to that proposed in humans and mouse model may trigger AGS through activation of TLR by α-Gal leading to production of proinflammatory cytokines and anti–α-Gal IgE response. As proposed for humans in response to tick saliva, basophils in zebrafish may be also recruited to attract T_H_2 cells producing IL-4 to the muscle inducing T_H_2-mediated IgE response to α-Gal. Basophils/eosinophils have been described in zebrafish and other fish species, but the functional role of these cells in immune response and allergy has not been previously characterized (Ainsworth, 1992; Bennett et al., 2001). The fact that *il1b* was the only gene upregulated in zebrafish suffering and dying of hemorrhagic anaphylactic-type reactions in response to tick saliva on day 2 and the regulation of this gene after red meat consumption suggesting a key role for this cytokine, which has been previously shown to promote adhesion of basophils, eosinophils, and neutrophils to human vascular endothelial cells (Bochner et al., 1991). Although a role for PGE_2_ during AGS has been proposed in humans (Cabezas-Cruz et al., 2017c), in the zebrafish model we did not find a correlation between this prostaglandin and the allergic response to tick saliva.

Based on the evidence obtained from studies in humans and the mouse and zebrafish animal models, the proposed mechanisms triggering the AGS involve TLR-mediated responses in both T_H_1 and T_H_2 cells with a role for basophils in this process (Figures 10B–D). The TLRs play a role in immune response by initiating signaling cascades that result in the recruitment of signaling adaptors such as MyD88 to trigger the formation of supramolecular organizing centers that coordinate various cellular responses such as translocation of Akr2/NF-κB and the activation of immune cells leading to the expression of proinflammatory cytokines and IFNs (Rosadini and Kagan, 2015). Pathogen-derived glycoproteins and glycolipids interact with TLRs with different outcomes. The TLR4 sensors bacterial lipopolysaccharides that activate the MyD88-dependent pathway resulting in Akr2/NF-κB activation leading to the production of IFN and proinflammatory cytokines (Perrin-Cocon et al., 2017). However, pathogen-derived glycolipids and glycoproteins can antagonize TLR-mediated response to interfere with cellular immune response (Hajishengallis and Lambris, 2011; Cochet et al., 2019). Basophil levels increase and infiltrate lesions after tick infestations contributing to acquired immunity and secretion of the histamine-repellent factor in tick-resistant animals (Karasuyama et al., 2018b; Tabakawa et al., 2018). Basophils have been also shown to activate T_H_2 IL-4–mediated responses (Karasuyama et al., 2018a), which may lead to acquired immunity to ticks and the high anti–α-Gal IgE antibody levels associated with the AGS (Kageyama et al., 2019). Additionally, basophils may attract T_H_2 cells to the tick bite site to induce intrinsic T_H_2 immunity-promoting adjuvant function of tick salivary components to enhance IgE response to α-Gal–containing tick proteins causing the AGS (Hilger et al., 2019; Kageyama et al., 2019).

## Conclusions

In this study, a new animal model was developed using zebrafish for the study of allergic reactions in response to tick salivary biogenic substances and red meat consumption. The observed allergic hemorrhagic anaphylactic-type reactions and abnormal behavior patterns may occur in response to toxic and anticoagulant biogenic compounds different from α-Gal present in tick saliva. Furthermore, host-derived and not only tick-derived molecules with α-Gal may be involved in the AGS (Platts-Mills et al., 2020). However, the results showed that only zebrafish previously exposed to tick saliva and fed on dog food developed hemorrhagic anaphylactic-type allergic reactions and/or abnormal behavior or feeding patterns with rapid desensitization and tolerance. These allergic reactions were associated with tissue-specific TLR-mediated responses in T_H_1 and T_H_2 cells with a possible role for basophils in the immune response to tick saliva. The results obtained in this proof-of-concept study support some of the previously proposed immune mechanisms triggering the AGS in humans and the α1,3-GalT-KO mouse model and provided evidence for different tissue-specific mechanisms also potentially involved in the AGS. These results support the use of the zebrafish animal model for the study of the AGS and other tick-borne allergies.

## Data Availability Statement

All datasets generated for this study are included in the article/Supplementary Material.

## Ethics Statement

The animal study was reviewed and approved by Ethics Committee on Animal Experimentation of the University of Castilla La Mancha (PR-2018-06-13) and the Counseling of Agriculture, Environment and Rural Development of Castilla La Mancha (ES130340000218).

## Author Contributions

JF and AC-C: conceptualization. JF: methodology, writing-original draft preparation, visualization, supervision, and project administration. MC, IP, and LM-H: validation. MC and JF: formal analysis. MC, IP, PA, SD-S, SA-J, LM-H, and MV: investigation. JF, MC, PA, SA-J, and AC-C: writing-review and editing. JF and MV: funding acquisition.

### Conflict of Interest

The authors declare that the research was conducted in the absence of any commercial or financial relationships that could be construed as a potential conflict of interest.
