# Supplementary material for: Allergic Reactions and Immunity in Response to Tick Salivary Biogenic Substances and Red Meat Consumption in the Zebrafish Model
Source: Front Cell Infect Microbiol. 2020 Mar 10;10:78. doi: 10.3389/fcimb.2020.00078 (PMC7075944; doi:10.3389/fcimb.2020.00078)
Supplement: Supplementary file 1 [file Data_Sheet_1.PDF]

Allergic reactions and immunity in response to tick salivary biogenic substances and red meat consumption in the zebrafish model

Marinela Contreras, Iván Pacheco, Pilar Alberdi, Sandra Díaz-Sánchez, Sara Artigas-Jerónimo, Lourdes Mateos-Hernández, Margarita Villar, Alejandro Cabezas-Cruz, José de la Fuente

Supplementary Materials:

Supplementary Figures 1-5

Supplementary Table 1

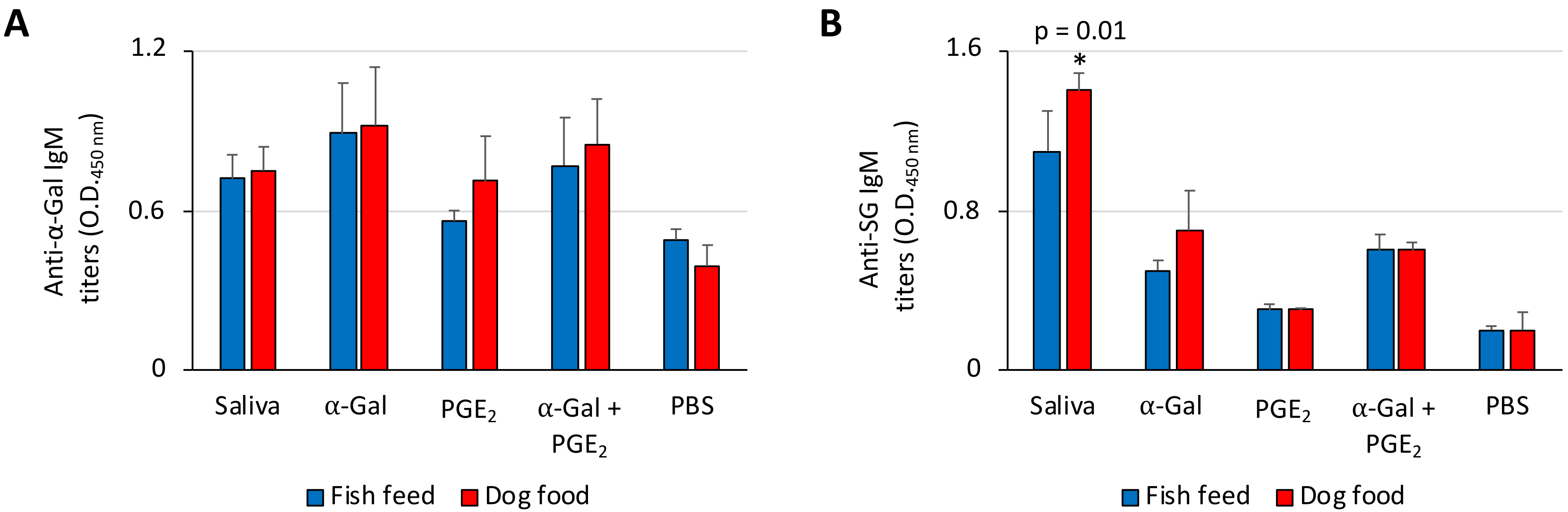

Supplementary Figure 1. Effect of feeding on zebrafish antibody response. The IgM antibody titers against (A) α-Gal and (B) tick salivary gland proteins (SG) were determined by ELISA, represented as the average + S.D. O.D. at 450 nm and compared between zebrafish fed with fish feed or dog food and treated with saliva, α-Gal, PGE_2, α-Gal + PGE_2 and PBS control by Student's t-test with unequal variance (*$p < 0.05$; N = 3-6).

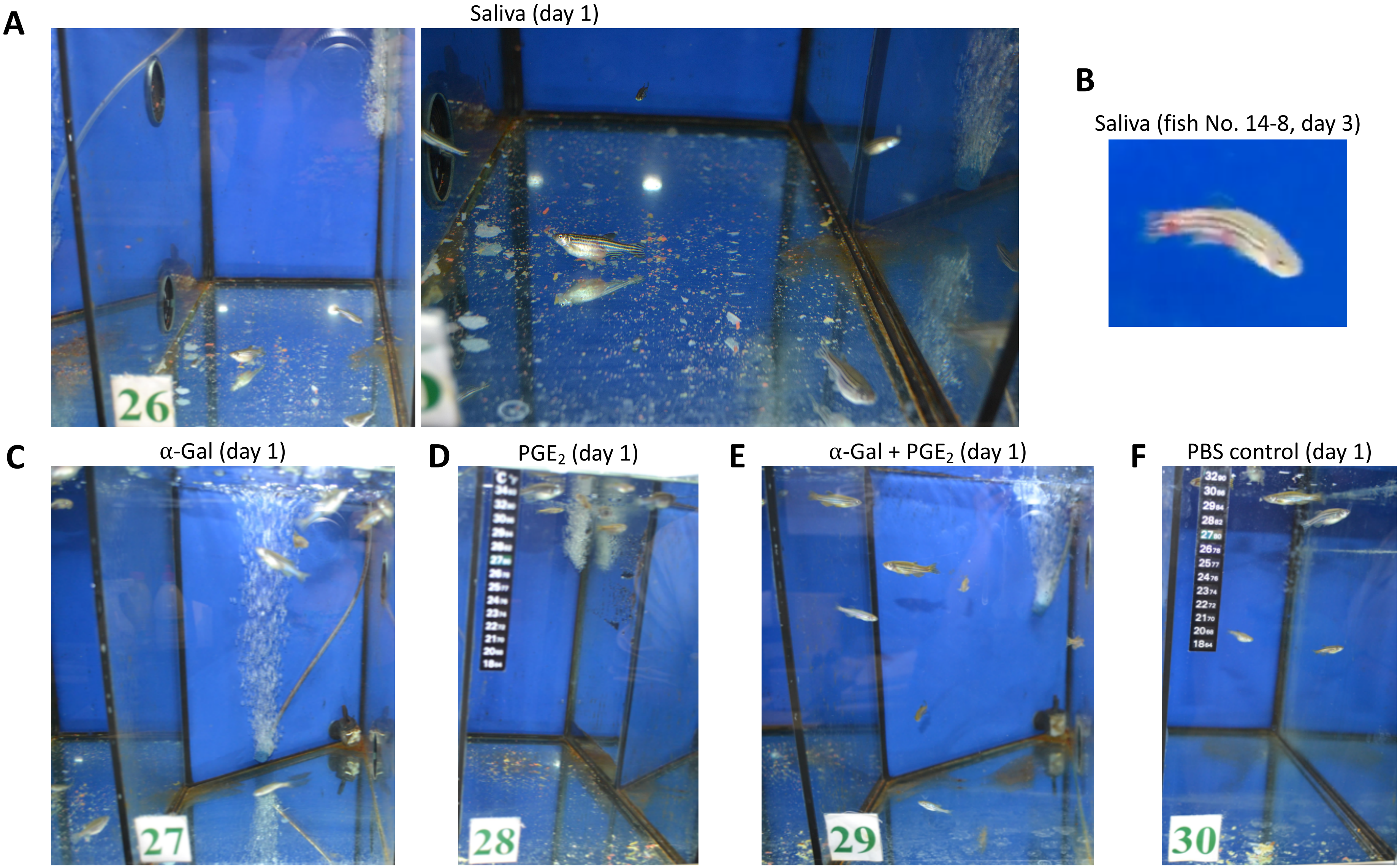

Supplementary Figure 2. Zebrafish injected with tick saliva develop abnormal behavior patterns. (A-F) Zebrafish behavior was recorded at day 1 after treatment. Abnormal behavior patterns consisting of low mobility and permanence at the bottom of the water tank was shown in (A) 3 zebrafish injected with tick saliva, (C) one zebrafish injected with α-Gal and (E) one zebrafish injected with α-Gal + PGE_2. (B) An abnormal zig-zag type swimming was observed at day 3 in fish No. 14-8 injected with tick saliva and fed with dog food (Supplementary Figure 3). Normal behavior patterns were seen in all zebrafish injected with (D) PGE_2 and (F) PBS.

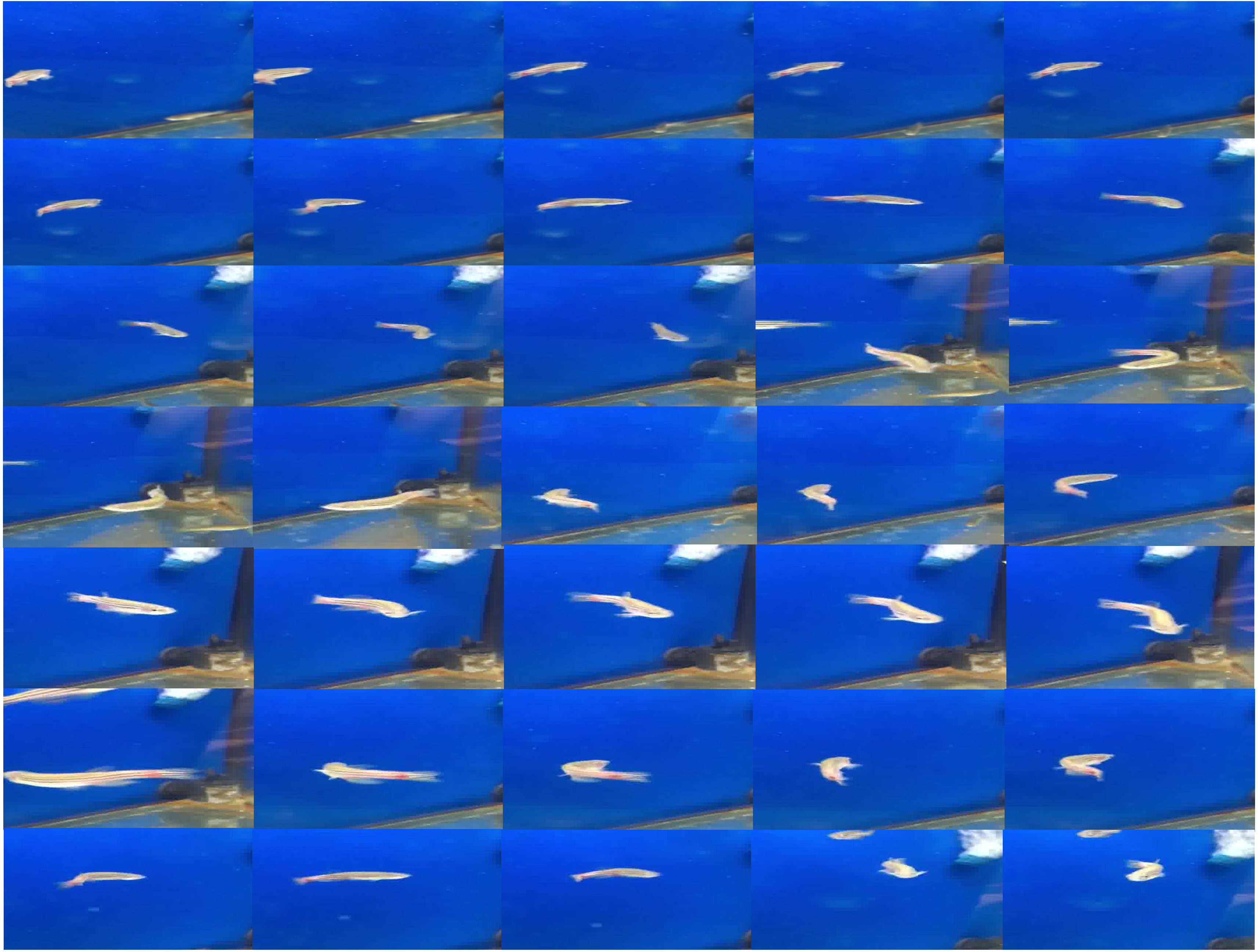

Supplementary Figure 3. Zebrafish injected with tick saliva and fed with dog food develop abnormal behavior patterns. Abnormal zig-zag type swimming at day 3 in zebrafish No. 14-8 injected with tick saliva and fed with dog food. Representative images were extracted from the video (https://youtu.be/ukud2TYN9sQ) using the Filezigzag online file conversion (https://www.filezigzag.com).

Saliva/Dog food

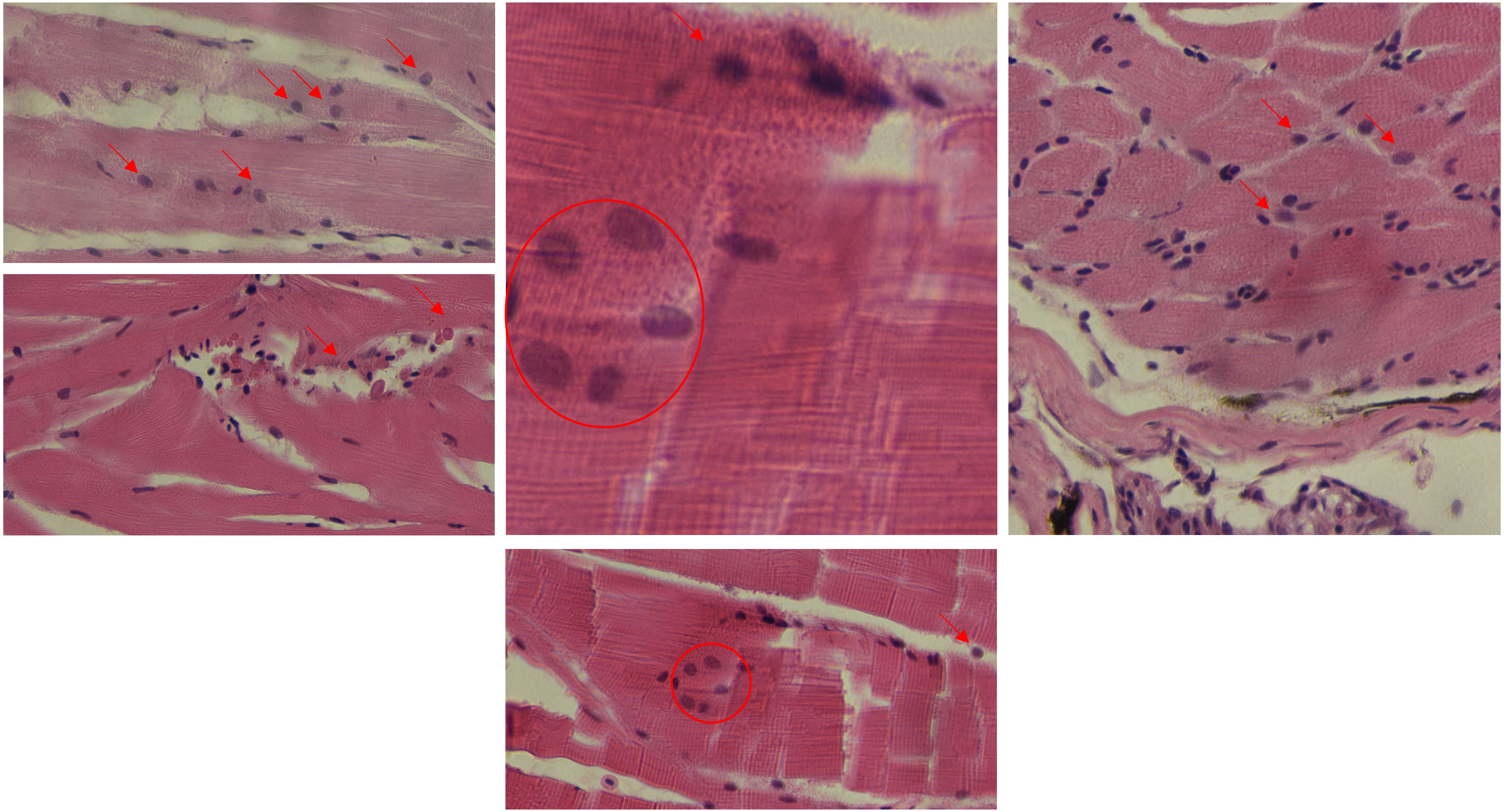

Granulocytes counts	Sample 1	Sample 2	Sample 3	Ave	SD
Saliva/Dog food	9	8	10	9.0	1.0

α-Gal/Dog food

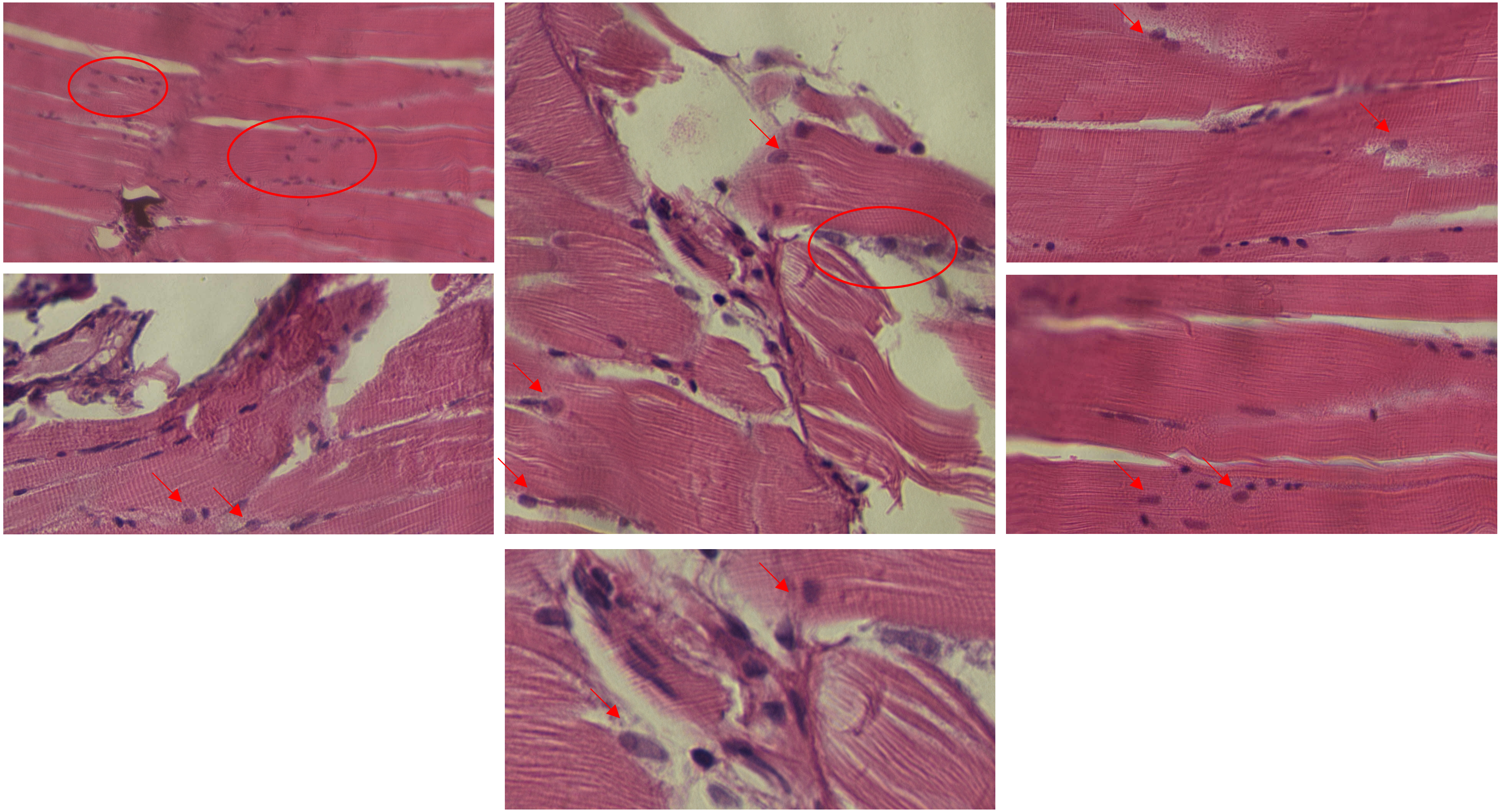

Granulocytes counts	Sample 1	Sample 2	Sample 3	Ave	SD
α-Gal/Dog food	3	2	2	2.3	0.6

PGE_2/Dog food

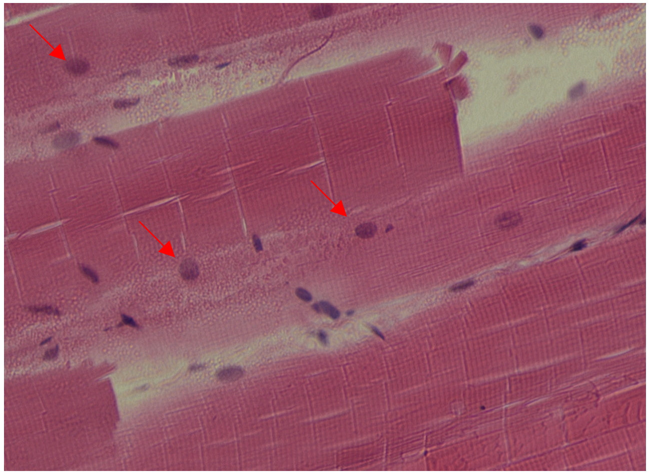

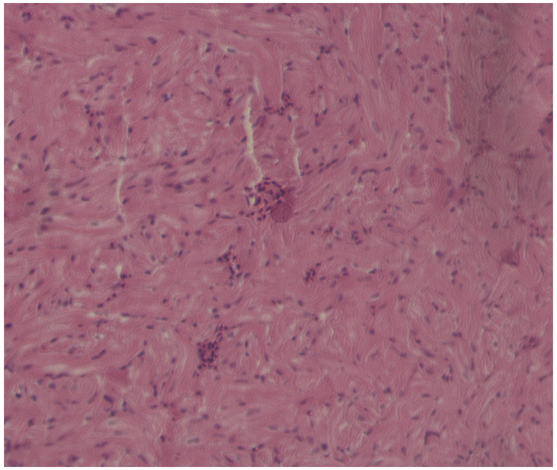

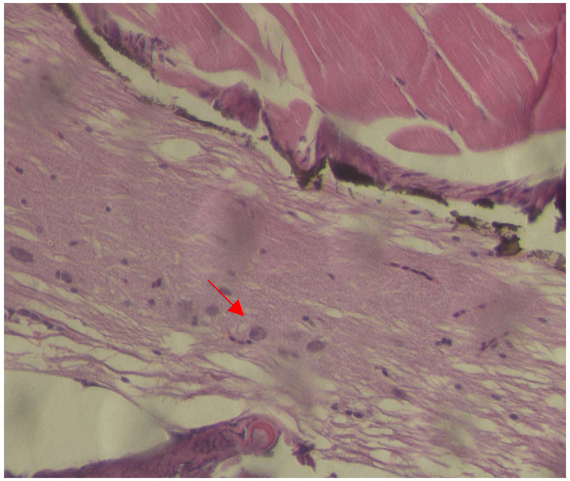

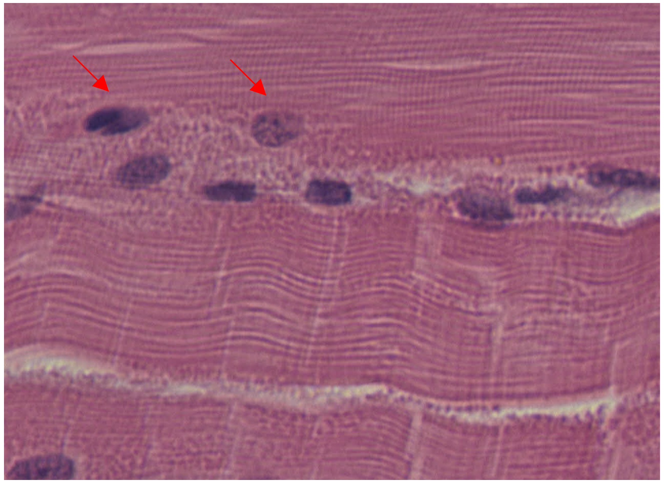

Granulocytes counts	Sample 1	Sample 2	Sample 3	Ave	SD
PEG2/Dog food	4	3	3	3.3	0.6

α-Gal + PGE_2/Dog food

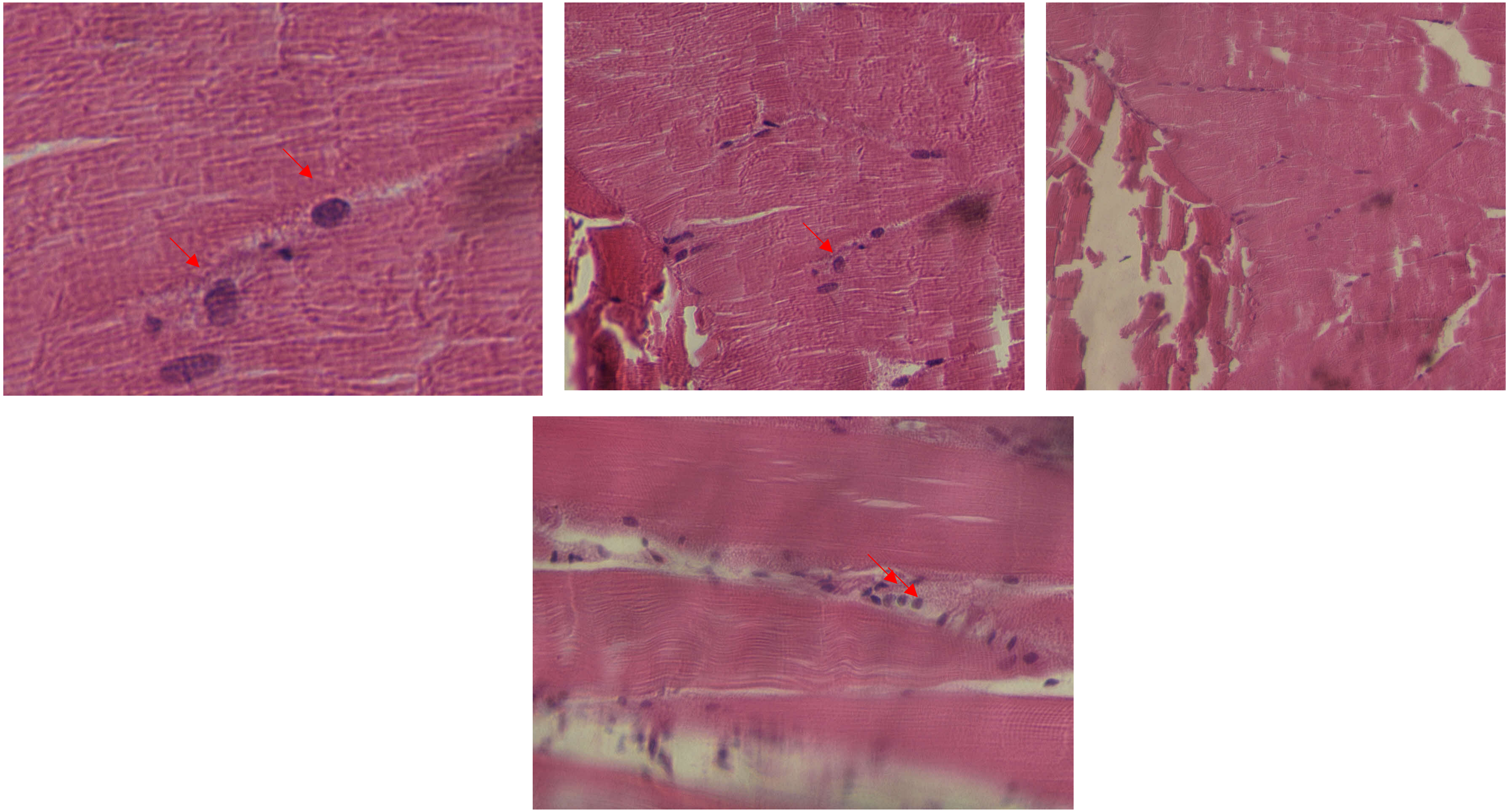

Granulocytes counts	Sample 1	Sample 2	Sample 3	Ave	SD
α-Gal+PGE2/Dog food	2	3	4	3.0	1.0

PBS/Dog food

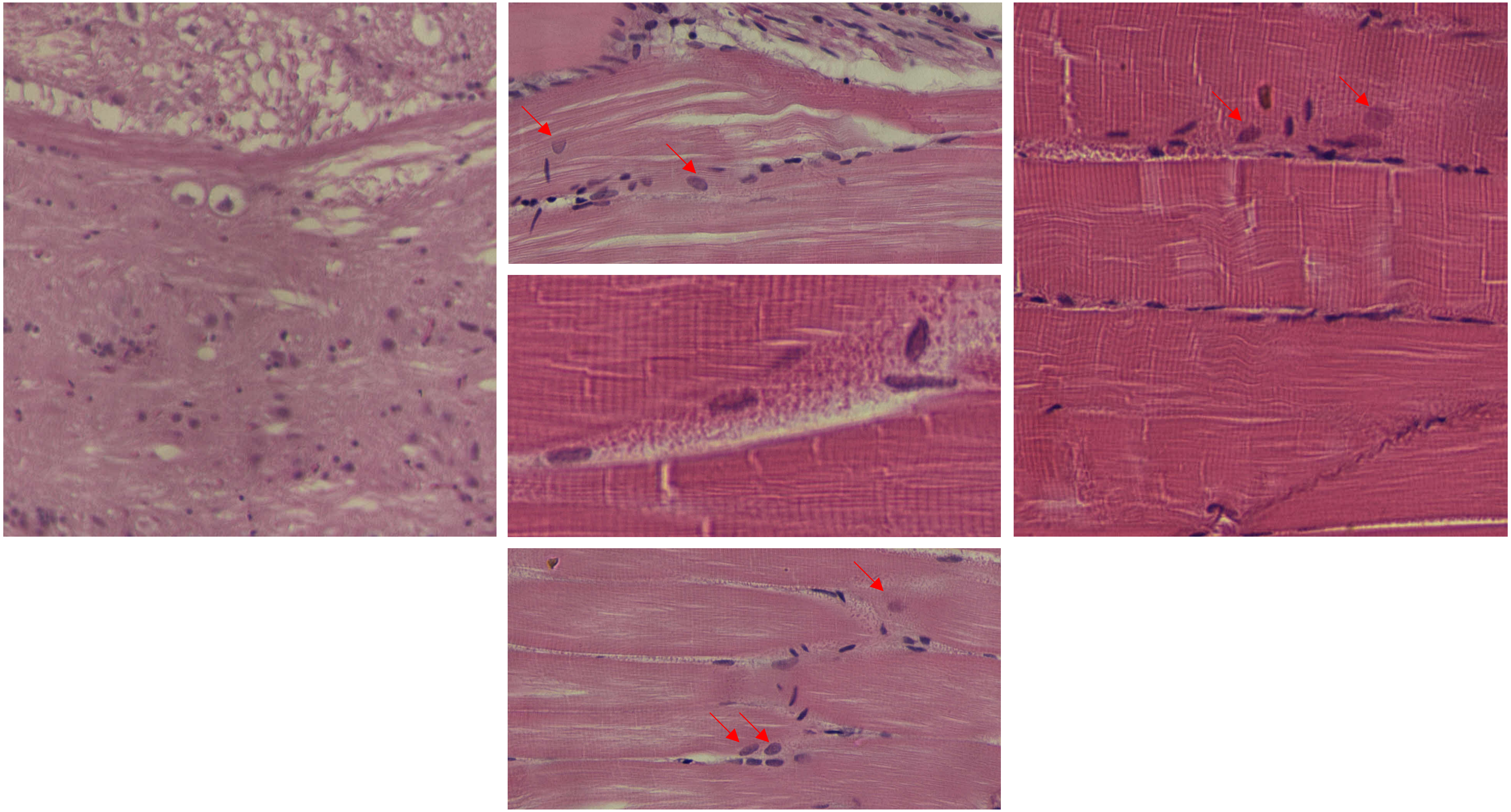

Granulocytes counts	Sample 1	Sample 2	Sample 3	Ave	SD
PBS/Dog food	2	3	3	2.7	0.6

Saliva/Fish feed

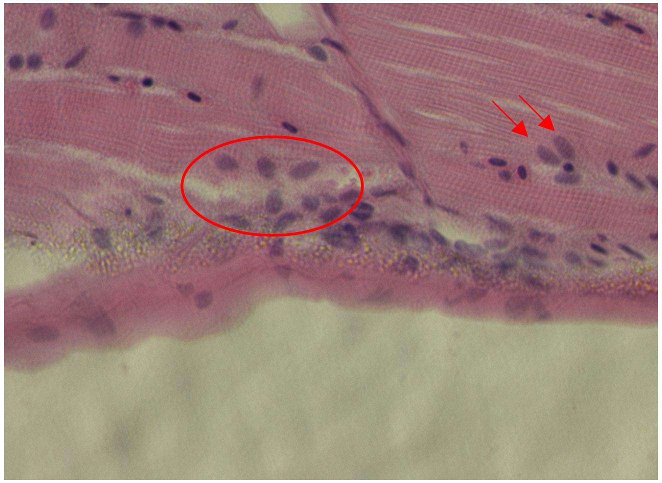
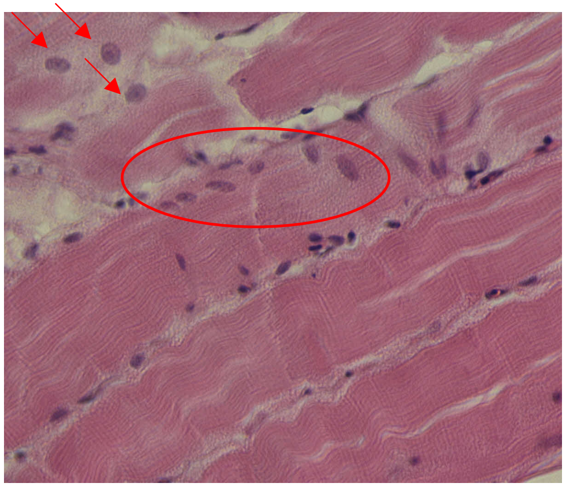
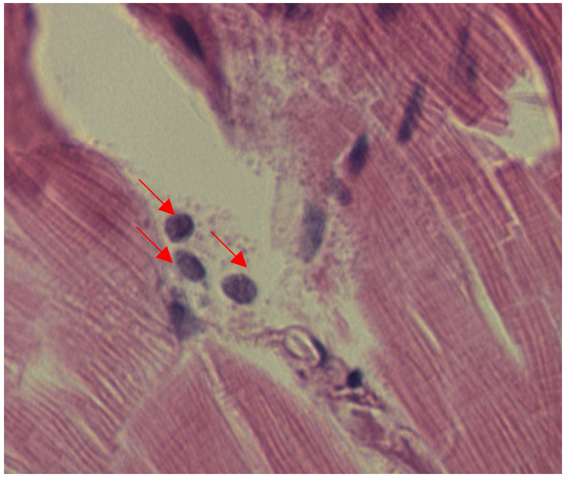
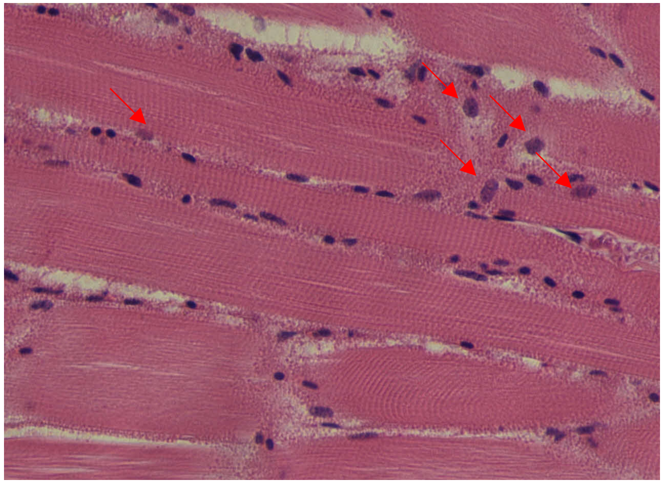

Granulocytes counts	Sample 1	Sample 2	Sample 3	Ave	SD
Saliva/Fish feed	8	9	9	8.7	0.6

α-Gal/Fish feed

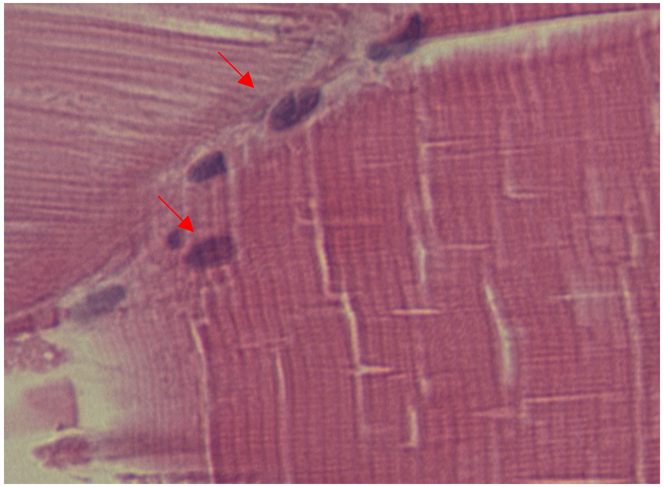

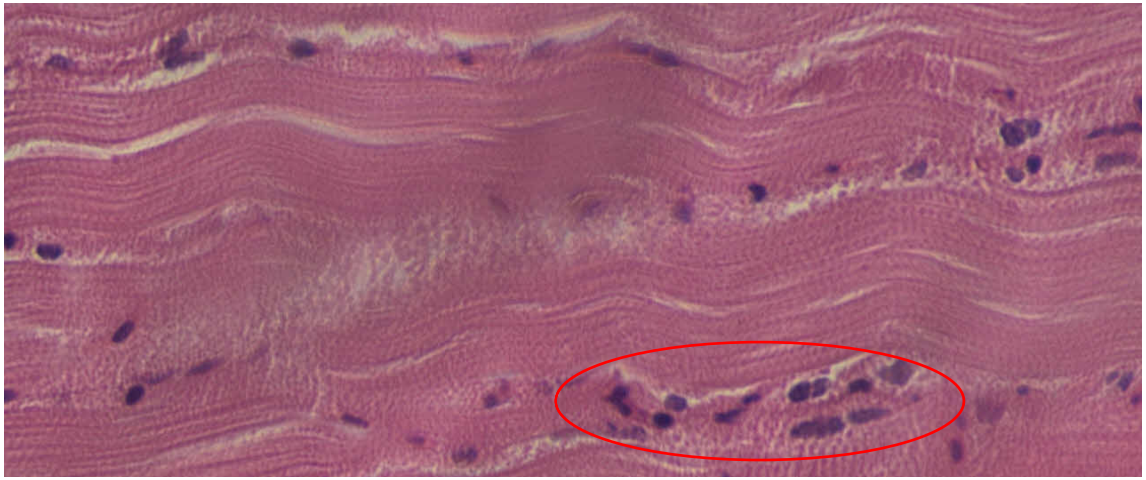

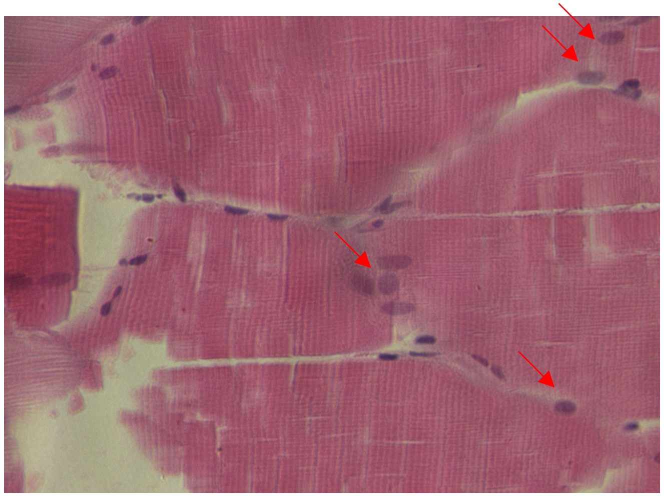

Granulocytes counts	Sample 1	Sample 2	Sample 3	Ave	SD
α-Gal/Fish feed	4	3	2	3.0	1.0

PGE_2/Fish feed

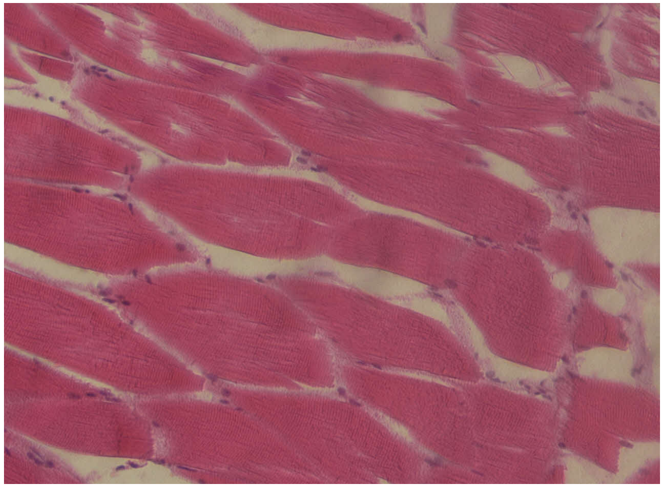

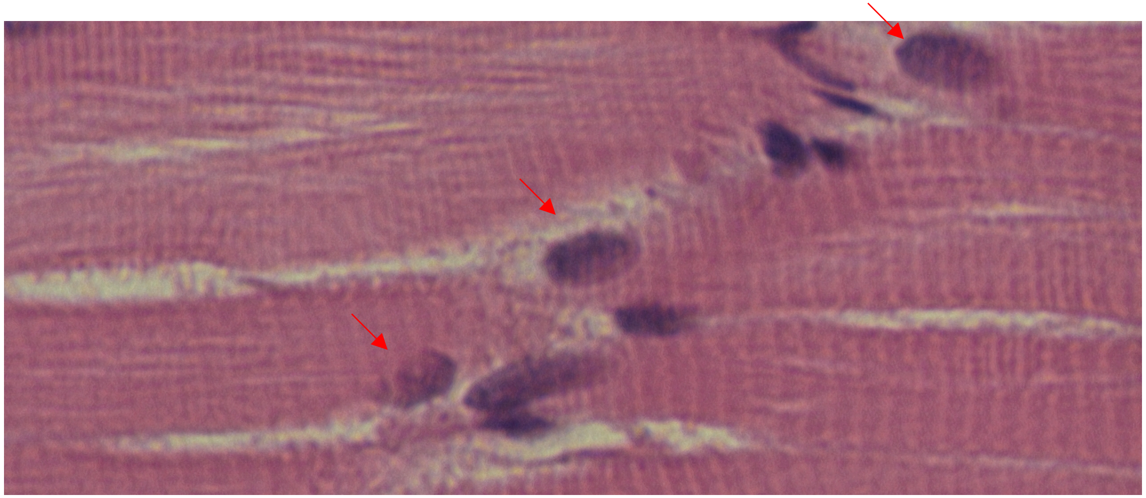

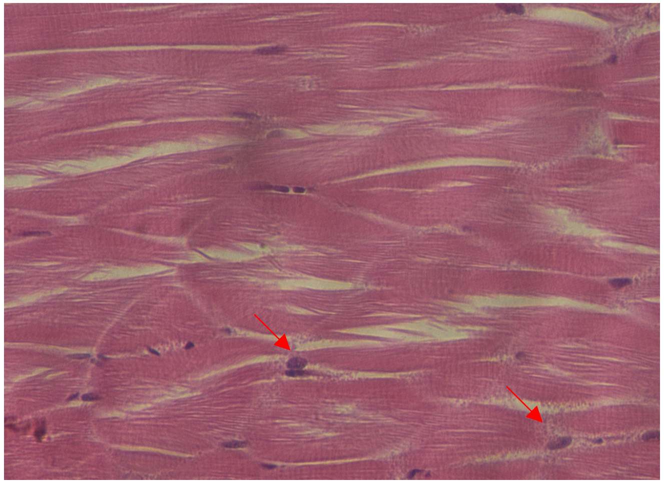

Granulocytes counts	Sample 1	Sample 2	Sample 3	Ave	SD
PGE2/Fish feed	2	3	4	3.0	1.0

α-Gal + PGE_2/Fish feed

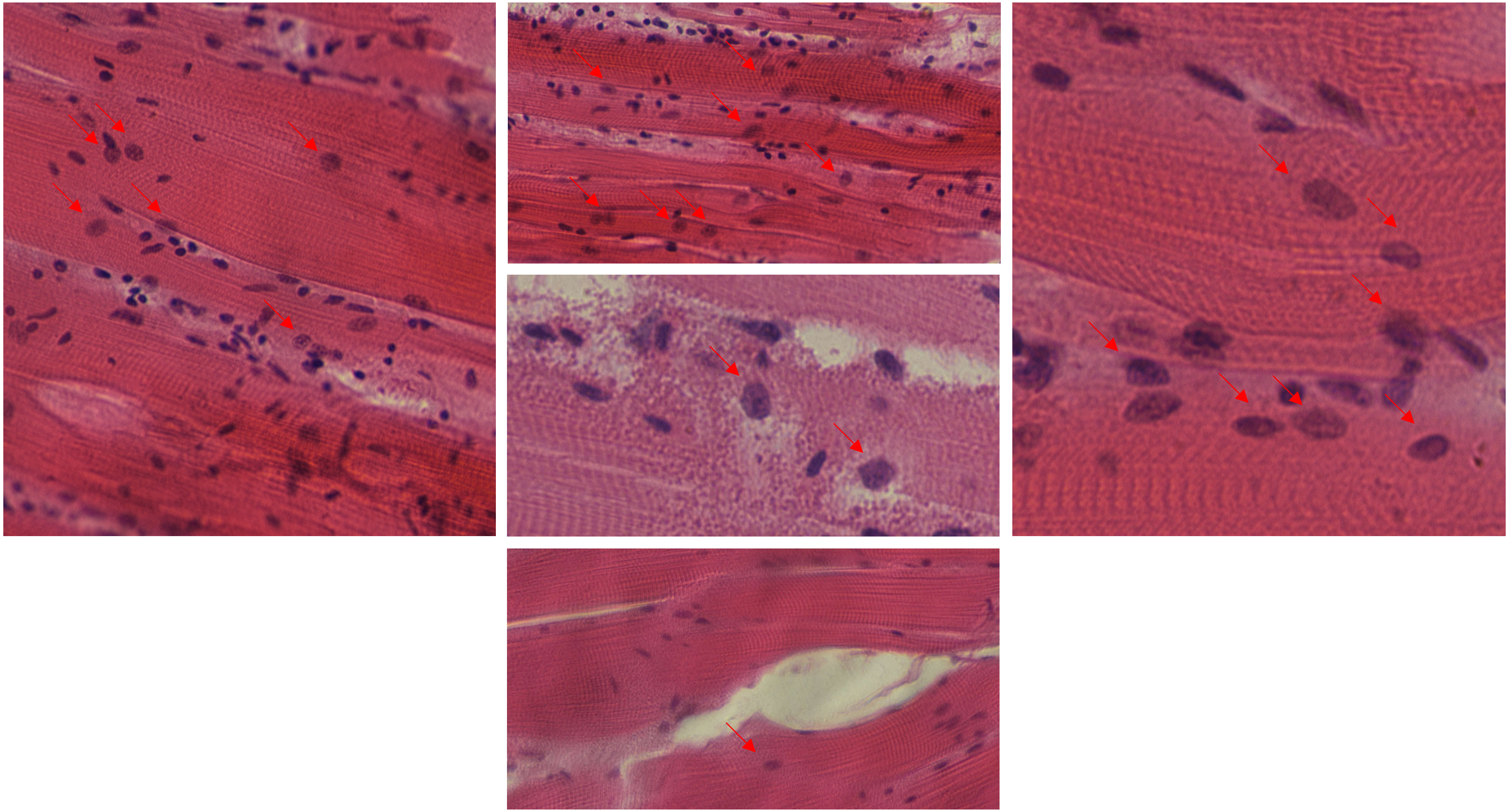

Granulocytes counts	Sample 1	Sample 2	Sample 3	Ave	SD
α-Gal+PGE2/Fish feed	4	2	4	3.3	1.2

PBS/Fish feed

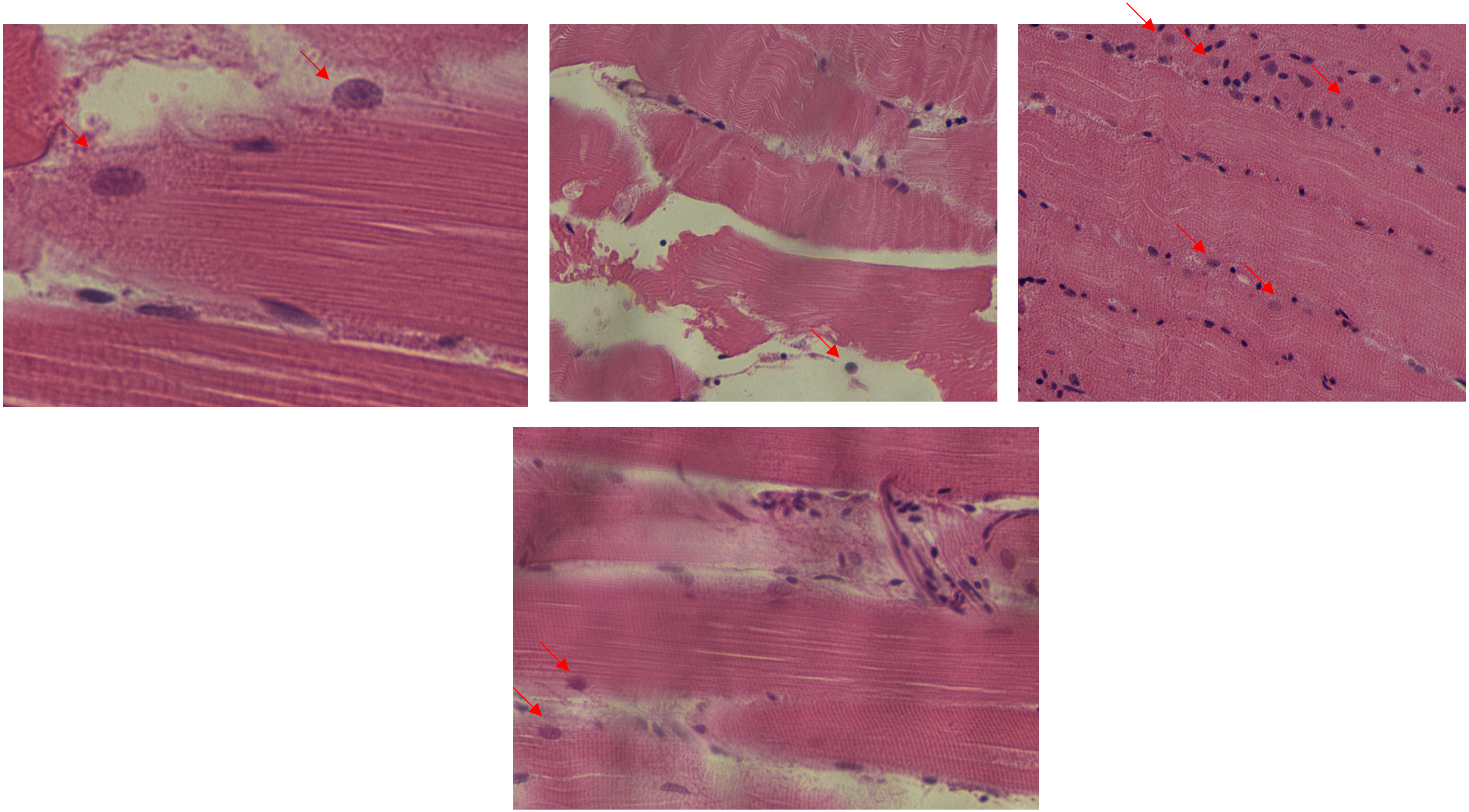

Granulocytes counts	Sample 1	Sample 2	Sample 3	Ave	SD
PBS/Fish feed	2	2	3	2.3	0.6

P (t-Test) Treatment vs. PBS

Saliva vs. PBS
0.00000002

α-Gal vs. PBS
0.34

PGE2 vs. PBS
0.06

α-Gal+PGE2 vs. PBS
0.09

P (t-Test) Dog vs. Fish feed

Saliva
0.32

α-Gal
0.19

PGE2
0.32

α-Gal+PGE2
0.36

PBS
0.26

Supplementary Figure 4. Granulocytes in the skeletal muscle of zebrafish. Representative images of granulocytes detected in muscle tissue sections stained with hematoxylin and eosin of zebrafish treated with saliva, α-Gal, PGE_2 or α-Gal + PGE_2 or the PBS control and fed with dog food or fish feed. The fields were randomly chosen, and granulocytes are indicated with arrows. Magnifications, 40X and 100X. The average counts of granulocytes were compared between fish treated with tick saliva, α-Gal or PGE_2 α-Gal + PGE_2 and PBS-treated controls and between fish fed on dog food or fish feed for each treatment by Student's t-test with unequal variance ($p < 0.05$; N = 3-6).

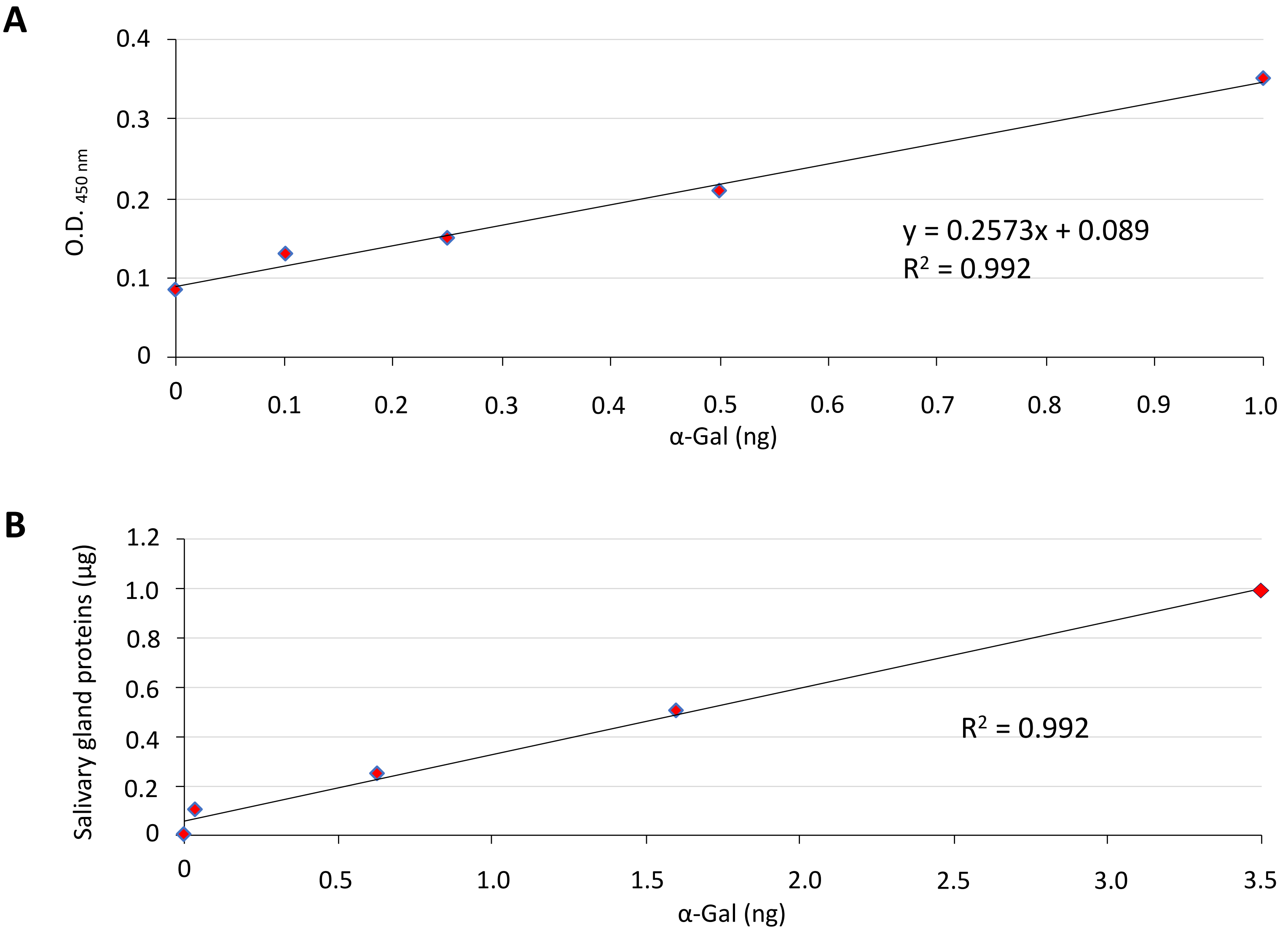

Supplementary Figure 5. ELISA test for characterizing α-Gal levels. The α-Gal levels were determined by ELISA using BSA-α-Gal and *R. sanguineus* salivary gland proteins. The average value of the blanks (wells without sample proteins; N = 5) was subtracted from all reads and the average of 9 replicates for each sample was used for analysis. (A) A calibration curve with 0.0 to 1.0 ng α-Gal and O.D. values at 450 nm was constructed using Microsoft Excel for Mac (v. 16.26) to convert ELISA reader values to α-Gal content per sample. (B) To further validate the calibration curve, a correlation was constructed between 0.0 to 3.5 ng α-Gal and 0.0 to 1.0 µg tick salivary gland proteins using Microsoft Excel for Mac (v. 16.26).

Supplementary Table 1. Oligonucleotide primers and conditions for qRT-PCR.

Gene (Accession No.)	Oligonucleotide primers	Annealing temperature
gapdh NM_001115114.1	Forward: 5´-CGTGGTGCCAGTCAGAACAT-3´	56 ºC
	Reverse: 5´-AGTCAGTGGACACAACCTGG-3´	
tlr2 NM_212812.1	Forward: 5´-TGAATGGGTCGAGGAGATTC-3´	56 ºC
	Reverse: 5´-CACAAAGTGCTCCGACAGAA-3´	
tlr4b NM_001131051.1	Forward: 5´-TCACCTGGACAGCAAGAATG-3´	56 ºC
	Reverse: 5´-CGATTGACTTCCCTGCTTGA-3´	
il1b NM_212844	Forward: 5´-GCATGTCCACATATGCGTCG-3´	58 ºC
	Reverse: 5´-GCTGGTCGTATCCGTTTGGA-3´	
il4 NM_001170740.1	Forward:5´-GTGAATGGGATCCTGAATGG-3´	56 ºC
	Reverse: 5´-TTCCAGTCCCGGTATATGCT-3´	
nfil3 NM_001004120.2	Forward: 5´-ATCACCAGGAGGCCCTAACT-3´	56 ºC
	Reverse: 5´-CTTTTCAAGCAGGCCACTTC-3´	
c3a NM_131243.1	Forward: 5´-ACGCTCTCTGGATTGAAACA-3´	56 ºC
	Reverse: 5´-TGCCTTCTTGCATGGCAATC-3´	
akr2 NM_213294.2	Forward: 5´-ACTATGGACTTCGATCCGCT-3´	56 ºC
	Reverse: 5´-GCTCTGTGGTGAGTGCTGAA-3´	
mxa NM_182942.4	Forward: 5´-AGTACCGGGGAAGAGAGCTA-3´	54 ºC
	Reverse: 5´-AAGGTGGCATGATTGTCTGT-3´	
ifn AJ544822	Forward: 5´-ATGAGAACTCAAATGTGGAC-3´	50 ºC
	Reverse: 5´-TTACACTCGAGGATTGAC-3´	
myd88 NM_212814.2	Forward: 5´-ATCGCCAGTGAGCTTATCGA-3´	56 ºC
	Reverse: 5´-GTCCAGAACCAGACCTGTGT-3´	
